# HOMAc: A Parameterization of the Harmonic Oscillator Model of Aromaticity (HOMA) That Includes Antiaromaticity

**DOI:** 10.1021/acs.joc.4c02475

**Published:** 2025-01-15

**Authors:** Enrique M. Arpa, Sven Stafström, Bo Durbeej

**Affiliations:** † Division of Theoretical Chemistry, IFM, Linköping University, 58183 Linköping, Sweden; ‡ Institute of Organic Chemistry, RWTH Aachen University, 52056 Aachen, Germany; § Division of Theoretical Physics, IFM, Linköping University, 58183 Linköping, Sweden

## Abstract

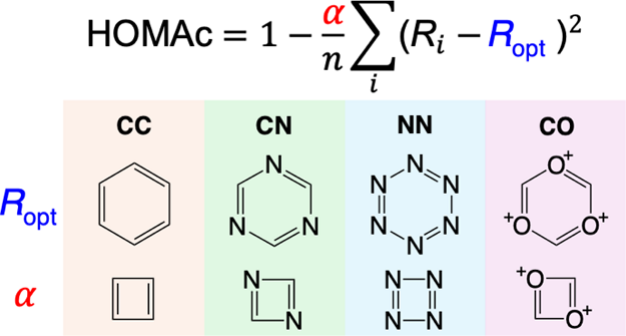

The harmonic oscillator model of aromaticity (HOMA) offers a straightforward route to quantifying aromaticity that requires no other information than the bond lengths of the conjugated ring in question. Given that such information is often readily obtainable from quantum-chemical calculations, it is pertinent to improve this parametrized model as much as possible. Here, a new version of HOMA is presented where, atypically, the corresponding parameters are derived from the actual bond lengths of both aromatic and antiaromatic (rather than nonaromatic) reference compounds, as calculated with a high-level method. The resulting model, which we denote HOMAc, covers CC, CN, NN, and CO bonds and is tested at eight different levels of theory for 45 (single-ring, multi-ring, carbocyclic, N,O-heterocyclic) molecules across the aromatic–antiaromatic spectrum. Thereby, it is found that HOMAc provides a description of aromaticity and antiaromaticity in better accord with magnetic, energetic, and π-delocalization-based reference data than does the standard parametrization of HOMA. Altogether, the results highlight the possibility to realize more reliable geometry-based probing of (anti)aromaticity with the use of HOMAc and with substantial freedom in the choice of quantum-chemical method.

## Introduction

While the aromaticity concept is ubiquitous in chemistry and readily applied to obtain qualitative assessments of both structures and chemical reactivities of cyclic, conjugated π-electron systems, more quantitative uses of this concept are challenged by the impossibility to measure or calculate aromaticity directly.^1−5^ For this reason, quantitative treatments of aromaticity typically employ indirect strategies where rather physicochemical properties associated with the manifestation of aromaticity are probed, such as diamagnetic ring currents, energetic stabilization or electron delocalization.^4,5^ Throughout the years, strategies of this kind have led to the formulation of many different types of calculable magnetic and energetic aromaticity indices, including the nucleus-independent chemical shift (NICS)^6,7^ and isomerization stabilization energy (ISE)^8^ indices, respectively. Other indices, such as the *para*-delocalization (PDI),^9^ aromatic fluctuation (FLU)^10^ and multicenter (MCI)^11^ ones, instead quantify aromaticity in terms of different measures of electron delocalization.^12,13^

A fourth category of indices are of geometric nature and utilize the fact that aromatic systems tend to show equalization of bond lengths.^14,15^ One particularly appealing feature of these indices is the ease with which their values can be obtained^16,17^ from experimentally determined (e.g., using diffraction techniques) or calculated (e.g., using quantum-chemical methods) molecular geometries. The first attempt to formulate such an index was made already in 1967 by Julg and François,^18^ who put forth an index exclusively applicable to carbocyclic systems of the form
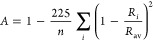
1where the summation runs over all *n* CC bonds in the ring with lengths *R*_*i*_, and *R*_av_ is the average of these lengths. The dimensionless constant 225, in turn, is a normalization parameter introduced to ensure that Kekulé’s hypothetical benzene structure, which is a common reference model of a nonaromatic system, assumes an *A* value of 0 when the bond lengths equal those of 1,3-butadiene. For a fully aromatic system, on the other hand, the *A* value becomes 1, as all *R*_*i*_ are then identical.

A major limitation of this so-called Julg index is that it attains a value of 1 for any carbocycle for which all CC bonds have the same lengths. Thus, it predicts cyclohexane to be just as aromatic as benzene! In a subsequent development, presented by Kruszewski and Krygowski in 1972 as the harmonic oscillator model of aromaticity (HOMA),^19^ these authors modified the Julg index by instead invoking an optimal CC bond length (*R*_opt_) achieved by a fully aromatic system. More specifically, *R*_opt_ was defined as the length of a bond at which the energy needed to extend it to a pure single bond (with length *R*_S_) equals the energy needed to compress it to a pure double bond (with length *R*_D_). Assuming these energies to depend harmonically on force constants and the ratio of force constants for pure single and double bonds to be 1:2, the *R*_opt_ parameter was calculated as
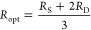
2

Taking experimentally determined^20^ CC bond lengths in ethane (1.524 Å) and ethylene (1.334 Å) as *R*_S_ and *R*_D_, respectively, the value of *R*_opt_ obtained from this formula (1.397 Å) is very close to the CC bond length of 1.398 Å predicted for benzene by neutron diffraction at 15 K.^21^ With this value, the HOMA index^19^ was defined as
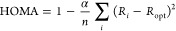
3where *n* and *R*_*i*_ have the same meaning as for the Julg index. α, in turn, is a normalization parameter determined in such a way that a model nonaromatic system–Kekulé’s benzene structure with the aforementioned ethane and ethylene CC bond lengths–shows a HOMA value of 0. In other words, α was determined based on the condition that
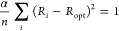
4for this system. Accordingly, denoting the ethane and ethylene CC bond lengths as *R*_S_ and *R*_D_, respectively, α was obtained as
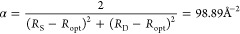
5

From the definition in eq 3, a fully aromatic system with all *R*_*i*_ equal to *R*_opt_ attains a HOMA value of 1, whereas the deviation of *R*_*i*_ from *R*_opt_ in a less aromatic system produces a smaller HOMA value.

In 1993, Krygowski presented a more general HOMA index applicable not only to carbocyclic π-electron systems, but also to heterocyclic compounds comprising up to seven different types of bonds (CC, CN, NN, CO, CP, CS and NO).^22^ Specifically, this index, which is often denoted HOMA93, takes the form
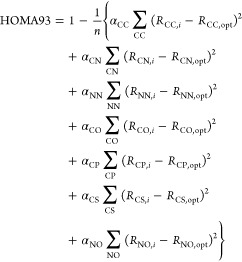
6where *n* is the total number of bonds in the cyclic structure and a bond between atoms X and Y are assigned *R*_XY,opt_ and α_XY_ values that are specific for that type of bond. For each bond type, the corresponding values were determined using experimental bond lengths of suitable reference systems, following the same approach as that outlined above. Henceforth in this work, the HOMA index is taken to refer to this 1993 parametrization. Besides being applicable to heterocycles, this model also made use of a different reference system from which to extract the experimental CC single- and double-bond lengths, replacing ethane and ethylene (1.524 and 1.334 Å)^20^ in the 1972 parametrization with 1,3-butadiene (1.467 and 1.349 Å).^23^ The rationale for this change is that, for every ring containing double or triple bonds, there will be some degree of π-electron delocalization, even if the ring is nonaromatic. Thus, an appropriate reference system should account for such delocalization. While the updated *R*_CC,opt_ parameter was only marginally affected by this change, decreasing from 1.397 to 1.388 Å, the updated α_CC_ parameter increased considerably, from 98.89 to 257.7 Å^–2^.^22^ In subsequent years, HOMA parameters have also been derived for a number of other types of bonds, such as BN,^24^ CB,^25^ CSe,^26^ COs^27^ bonds and CC bonds in radical species,^28^ or used in applications of the HOMA index as a more general geometric similarity index.^29^

In 2010, Raczyńska and co-workers presented a new HOMA-like index, parametrized for CC, CN and CO bonds, that they termed the harmonic oscillator model of electron delocalization (HOMED).^30^ Although this index takes the same exact form as HOMA, it was developed in somewhat different ways, with the goal to improve the description of electron delocalization in heterocycles.^30^ First, the *R*_opt_ and α parameters were not derived from experimental bond lengths, but from bond lengths calculated with quantum-chemical methods. Given that HOMA/HOMED indices are often evaluated using geometries obtained computationally, this approach would eliminate errors due to intrinsic discrepancies between calculated and experimental geometries. Moreover, the *R*_opt_ parameters were not obtained by first choosing appropriate reference systems for extracting ideal CC/CN/CO single- and double-bond lengths, and then taking weighted averages of the two in the vein of eq 2. Instead, the *R*_opt_ parameters were obtained directly as the actual bond lengths of suitably chosen reference systems: benzene for CC bonds, 1,3,5-triazine for CN bonds and protonated carbonic acid for CO bonds. The corresponding α parameters, however, were still determined in the “traditional” HOMA way, using ideal CC/CN/CO single- and double-bond lengths (but employing more elaborate formulas than eq 5 in some cases).

Some of the ideas of Raczyńska and co-workers were subsequently continued by Frizzo and Martins, who considered a larger set of different bonds (CC, CN, NN, CO, CS, NO and NS).^31^ Contrary to Raczyńska and co-workers, however, these researchers based their model exclusively upon experimental bond lengths taken from X-ray and neutron diffraction measurements.^32^

In our own work in this field, which has partly been driven by the possibility to use the concepts of excited-state aromaticity and antiaromaticity^33−42^ as rational design tools in photophysics and photochemistry,^43−47^ we have recently discussed the need to be able to apply HOMA to molecules in excited states with the same expected accuracy as HOMA shows when applied to molecules in their electronic ground state (S_0_).^48^ In particular, we have presented the first-ever parametrization of HOMA tailored specifically to the lowest triplet ππ* excited state (T_1_).^48^ Denoting it the harmonic oscillator model of excited-state aromaticity (HOMER) and covering CC, CN, NN and CO bonds, this parametrization has a number of unique features.^48^ For example, both the *R*_opt_ and the α parameters are derived directly from the actual bond lengths of pertinent reference systems, using one set of systems for the former and another set of systems for the latter parameters, and parametrizing each bond type XY according to
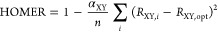
7Here, the *R*_XY,opt_ parameters are obtained as the bond lengths *R*_XY,*i*_ of reference systems known to be *aromatic* in T_1_, which means that these reference systems show HOMER values of exactly 1. Moreover, the α_XY_ parameters are derived from the bond lengths *R*_XY,*i*_ of reference systems known to be *antiaromatic* in T_1_, using the condition that
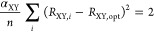
8which implies that the corresponding reference systems have HOMER values of exactly –1. Accordingly, antiaromaticity is explicitly included in the parametrization, which is another unique feature. Indeed, the goal of the α parametrization for HOMER that a model antiaromatic system should have a HOMER value close to –1, is very different from the goal of the α parametrization for HOMA that a model nonaromatic system should have a HOMA value close to 0. This means that the range of aromatic character included in the parametrization is much broader for HOMER (from antiaromatic to aromatic) than for HOMA (from nonaromatic to aromatic). Given the many recent examples of how photochemical reactivity is controlled by relief of excited-state antiaromaticity,^45−47,49−53^ this characteristic is likely to make HOMER a better tool for studying such mechanisms. Finally, a third distinguishing feature of HOMER is that the parametrization is based on calculated bond lengths as obtained with a gold-standard method (CASPT2, the ab initio multiconfigurational complete active space second-order perturbation theory method^54^), which is different from the use of a more approximate density functional theory (DFT) method in the parametrization of the aforementioned HOMED index by Raczyńska and co-workers.^30^

Notably, comparing the performances of HOMER and HOMA relative to reference data in the form of calculated NICS values, which are widely considered to be among the most dependable measures of aromaticity and antiaromaticity,^55,56^ it was then found that HOMER provides a much more accurate description of aromaticity and antiaromaticity in the T_1_ state than HOMA.^48^ In this light, it is of natural interest to employ the same exact strategies that governed the development of HOMER to obtain a new parametrization of HOMA that similarly improves the description of aromaticity and antiaromaticity in the S_0_ state. In this work, such a parametrization, which we have termed HOMAc (where “c” stands for “computational”), is presented and extensively tested for 45 molecules across the aromatic-antiaromatic spectrum. From these tests, involving eight different quantum-chemical methods and (following widespread recommendations^57^) reference data covering several different manifestations of aromaticity (magnetic, energetic and electronic), it is concluded that HOMAc enables more reliable probing of aromaticity and antiaromaticity in the S_0_ state than the standard 1993 parametrization of HOMA.^22^ Altogether, the results highlight the continued importance and improvability of geometry-based aromaticity indices.

## Results and Discussion

### Parametrization of HOMAc

**Figure 1:**
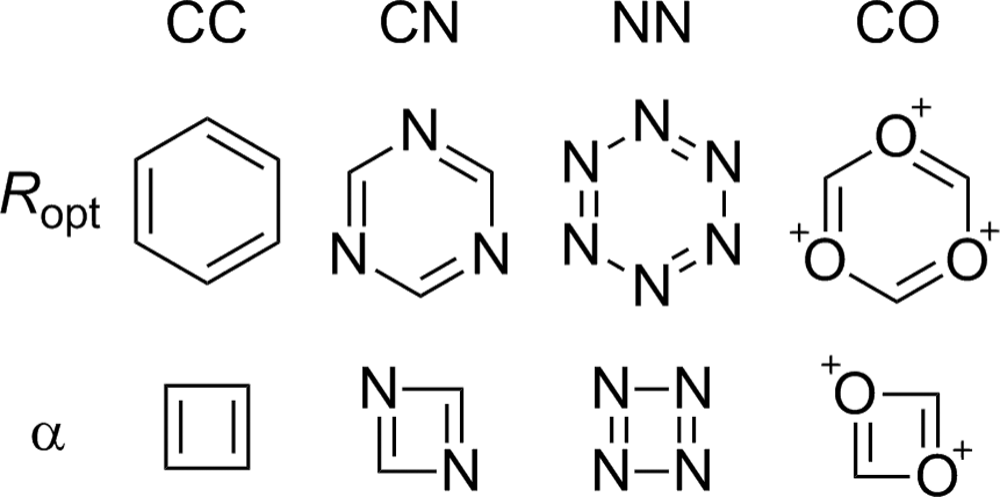
Reference systems used for the parametrization of HOMAc.

In the development of the HOMER index, the *R*_XY,opt_ parameters for the four types of bonds considered were obtained as CASPT2 bond lengths *R*_XY,*i*_ of four different reference systems, each known to be aromatic in T_1_ and featuring a ring with only CC (cyclobutadiene), CN, NN or CO bonds (aza- and oxo-analogues of cyclobutadiene), respectively.^48^ With the *R*_XY,opt_ parameters in hand, the corresponding α_XY_ parameters were then derived from CASPT2 bond lengths *R*_XY,*i*_ of four different reference systems conversely known to be antiaromatic in T_1_, using the condition encapsulated by eq 8.^48^ Specifically, the α_XY_ parameters were derived using benzene for the CC bond, and aza- and oxo-analogues of benzene for the CN, NN and CO bonds.^48^ For the current development of the HOMAc index, in turn, the associated *R*_XY,opt_ and α_XY_ parameters were obtained in exactly the same way as they were for HOMER, except that the reference systems from which the HOMER *R*_XY,opt_ and α_XY_ parameters were derived, were now used to calculate the HOMAc α_XY_ and *R*_XY,opt_ parameters, respectively. Importantly, this procedure ensures that HOMAc values calculated for the S_0_ state can be compared, in a balanced way, with HOMER values calculated for the T_1_ state. Also, this procedure follows naturally from the discovery by Baird that the (anti)aromatic character of annulenes with 4*n* and 4*n* + 2 π-electrons in the S_0_ state, as stated by Hückel’s rule, is the opposite of their (anti)aromatic character in the T_1_ state.^33^ Accordingly, the parametrization of HOMAc made use of the reference systems shown in Figure 1. As before, the parametrization was done by optimizing the S_0_ geometries of the reference systems with the CASPT2 method in combination with the large cc-pVQZ basis set.^58^ Without exception, from frequency calculations carried out at the same level of theory, the resulting geometries were found to be potential-energy minima.

The resulting HOMAc parameters are presented in Table 1, which also includes the corresponding HOMA^22^ (1993 version) and HOMER^48^ parameters. Before comparing the different sets of parameters, it should be mentioned that the use of four-membered reference systems in deriving the HOMAc α_XY_ parameters is natural in that these specific systems are prototypical S_0_ antiaromatic species. At the same time, these systems are strained, which is potentially negative for the aromaticity-probing ability of HOMAc. However, since we will show that HOMAc consistently achieves better agreement with other aromaticity indices than HOMA does, strain cannot play a role in this regard. As for comparing HOMAc to HOMA, the difference between the two parametrizations is mainly manifested in the α_XY_ values, which is expected as these are derived based on different conditions for the two indices (HOMAc = −1 for a model antiaromatic system and HOMA = 0 for a model nonaromatic system). The *R*_XY,opt_ values, on the other hand, are more similar, which is in accord with them being derived with the same goal in the two parametrizations (HOMAc = HOMA = 1 for a model aromatic system). Indeed, with the exception of the CO bond, HOMAc and HOMA have *R*_XY,opt_ values that are similar to within 0.7% or less. For the CO bond, the difference is larger (∼4%), with the HOMAc/*R*_CO,opt_ value exceeding the HOMA/*R*_CO,opt_ value by 0.05 Å and almost coinciding with the HOMAc/*R*_NN,opt_ value. This similarity of CO and NN bonds is not present in HOMA.

**Table 1:** HOMAc Parameters and the Corresponding HOMA and HOMER Parameters^a^

bond	HOMAc S_0_^b^		HOMA S_0_^b^		HOMER T_1_^b^	
	*R*_opt_ (Å)	α (Å^–2^)	*R*_opt_ (Å)	α (Å^–2^)	*R*_opt_ (Å)	α (Å^–2^)
CC	1.392	153.37	1.388	257.70	1.437	950.74
CN	1.333	111.83	1.334	93.52	1.390	506.43
NN	1.318	98.99	1.309	130.33	1.375	187.36
CO	1.315	335.16	1.265	157.38	1.379	164.96

a HOMA parameters taken from ref (22) and HOMER parameters taken from ref (48).

b State for which the model is parametrized.

### Comparing HOMAc and HOMA

**Figure 2:**
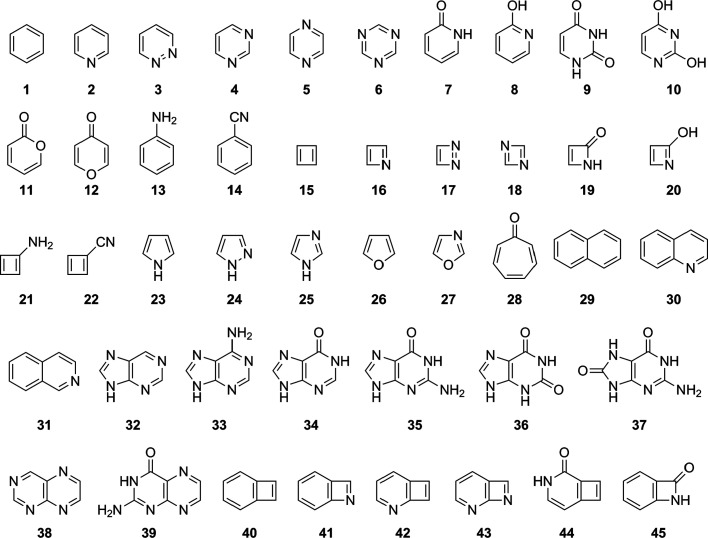
Compounds used to assess the performances of the HOMAc and HOMA indices.

In order to assess how well HOMAc performs compared to HOMA, both indices were used to probe the (anti)aromatic character of the 45 compounds shown in Figure 2. These molecules, which cover a large portion of the aromatic-antiaromatic spectrum, comprise both single-ring and multi-ring carbocyclic and N,O-heterocyclic systems of varying sizes and with different substituents. Their HOMAc and HOMA values (of individual rings for the case of multi-ring systems) were calculated with the use of the parameters in Table 1 and expressions analogous to eq 6. In order to realize a broad assessment, the requisite geometry optimizations were carried out with eight different quantum-chemical methods. Besides CASPT2, these included the ab initio Hartree–Fock (HF), Mo̷ller-Plesset second-order perturbation theory (MP2) and coupled-cluster singles and doubles (CCSD)^59^ methods, as well as DFT methods rooted in the generalized gradient approximation (GGA): M06-L (a meta-GGA),^60^ M06-2X (a global hybrid meta-GGA),^61^ B3LYP (a global hybrid GGA) and ωB97X-D (a range-separated hybrid GGA).^62^ All geometry optimizations were performed with the medium-sized cc-pVDZ basis set,^58^ which is a typical choice for such calculations (notice here that the aim of this work is not to calculate maximally accurate aromaticity indices for the compounds in Figure 2 using the largest possible basis set). From a compatibility viewpoint, another reason why it is sensible to use the cc-pVDZ basis set is to ensure that the performance of HOMAc is tested in exactly the same way as that of HOMER was tested in our previous work.^48^

Regarding the inclusion of CASPT2 among the quantum-chemical methods employed, this does not reflect that a treatment of multireference effects is needed to describe the compounds under investigation. Instead, the use of CASPT2 is motivated by our goal to also assess the transferability of the CASPT2-based HOMAc parametrization to calculations with other methods, which requires CASPT2 calculations as reference.

As a first test, HOMAc and HOMA values were compared with NICS_*zz*_ values (corresponding to the *zz*-component of the magnetic shielding tensor^7^) calculated with a set of methods (and the cc-pVDZ basis-set) as similar as possible to those with which the HOMAc and HOMA values were calculated. Specifically, while the HOMAc and HOMA values obtained with the HF, MP2, M06-L, M06-2X, B3LYP and ωB97X-D methods were compared with NICS_*zz*_ values calculated with exactly the same methods, the HOMAc and HOMA values obtained with the CASPT2 and CCSD methods were instead compared with NICS_*zz*_ values calculated with the complete active space self-consistent field (CASSCF)^63^ and MP2 methods, respectively. This procedure is motivated by the lack of a software for calculating NICS values with the high-level CASPT2 and CCSD methods. In the same vein, the main reason why HOMAc and HOMA values were not calculated at the gold-standard CCSD(T) level^64,65^ is not the higher cost of CCSD(T) relative to CCSD. Rather, it is the fact that while CCSD-level HOMAc and HOMA values are naturally comparable to MP2-level NICS values, a similarly balanced comparison of CCSD(T)-level HOMAc and HOMA values to NICS is currently not realizable.

The choice to assess the performances of HOMAc and HOMA relative to reference data in the form of NICS_*zz*_ values is sensible in light of the wide belief that NICS is a reliable aromaticity index.^55,56^ Furthermore, there are many observations that HOMA-based indices are well-correlated with NICS-based ones,^1,14,66−69^ despite that the two indices probe entirely different facets of aromaticity (bond-length equalization and induced ring currents in an external magnetic field, respectively). In addition to calculating the NICS_*zz*_ values at 1 Å distances above the geometric centers of the single-ring systems (or 1 Å above the geometric center of each ring of the multi-ring systems), which is an established procedure^7^ yielding values henceforth denoted NICS_*zz*_(1), distances of 0 (NICS_*zz*_(0)) and 2 Å (NICS_*zz*_(2)) were also considered. This serves to minimize the effect of any bias introduced by the specific choice of distance.^70^

**Figure 3:**
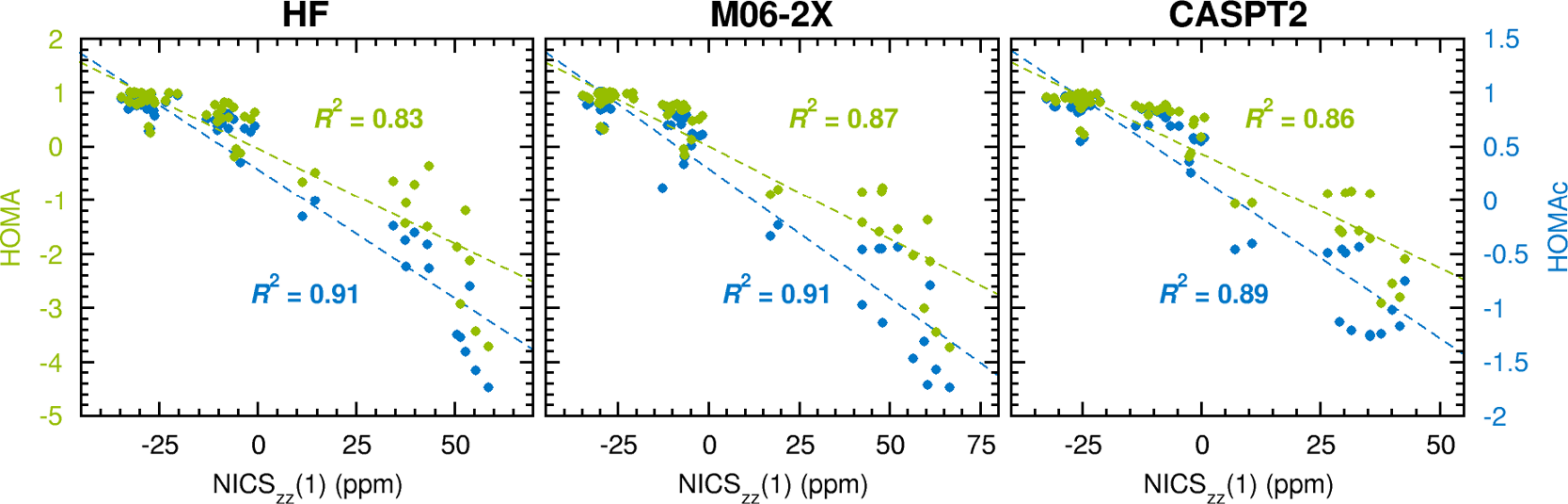
Linear correlations to NICS_*zz*_(1) values achieved by HOMAc (blue font) and HOMA values (green font) calculated at the HF, M06-2X and CASPT2 levels of theory.

The HOMAc and HOMA vs NICS_*zz*_(1) comparison is presented in Figure 3 (for HOMAc and HOMA values calculated with the HF, M06-2X and CASPT2 methods) and in Figure S1 of the Supporting Information (for HOMAc and HOMA values calculated with all the other methods). The comparisons with the NICS_*zz*_(0) and NICS_*zz*_(2) data, in turn, are presented in Figures S2 and S3 of the Supporting Information (these comparisons are done exclusively for HOMAc and HOMA values calculated with the HF, M06-2X and CASPT2 methods). All raw data from the HF, M06-2X and CASPT2 calculations are summarized in Tables S1–S3 of the Supporting Information.

Noting that negative/positive NICS values typically reflect aromaticity/antiaromaticity^6^ and quantifying the correlations with NICS data that HOMAc and HOMA achieve by *R*^2^ values from linear-regression analyses, our first focus is the performance of HOMA. Here, it is interesting to see from Figures 3 and S1 that HOMA fares rather well, attaining *R*^2^ values that range from 0.82 to 0.87. The correlations of HOMA with the NICS_*zz*_(0) and NICS_*zz*_(2) data are of similar magnitudes, 0.78–0.83 (Figure S2) and 0.79–0.83 (Figure S3), respectively. Overall, these results corroborate the aforementioned observations^1,14,66−69^ that HOMA is a viable alternative to the highly rated^55,56^ NICS index in probing aromaticity, especially considering the greater ease with which HOMA is calculated.

At this stage, it is important to clarify that different aromaticity indices are not necessarily *linearly* proportional to each other.^71−74^ However, in support of the current procedure to exclusively explore linear correlations, it has been demonstrated that energetic, geometric and magnetic indices (including HOMA and NICS) generally show significant collinearity, provided that the set of molecules studied is sufficiently varied in terms of the aromatic, nonaromatic and antiaromatic character of the molecules.^75^ Clearly, herein, this “condition” is satisfied. Moreover, given that NICS is the main reference index for the testing of HOMAc and HOMA in this work, further support for our procedure can be derived from the many previous studies that have documented strong linear correlations between HOMA and NICS for a wide variety of systems,^66−68,76^ often with *R*^2^ values larger than 0.90.

Despite the positive results, HOMA has a flaw in that antiaromaticity is not included in the parametrization. Besides possibly compromising the description of antiaromatic systems, this also means that there is no ideal antiaromatic reference value that can be used for assessing the HOMA values of such systems, in the same way that the HOMA values of aromatic systems can always be compared in terms of how close they are to 1. Thus, while antiaromatic systems show markedly negative HOMA values, it is not immediately clear what a comparison of such values implies. For example, the most negative HOMA values calculated with the three methods in Figure 3 are those of the archetypal antiaromatic cyclobutadienes **15** (between –2.8 and –3.7), **21** (between –2.9 and –3.0) and **22** (between −2.6 and −3.5). HOMAc, on the other hand, does include antiaromaticity in the parametrization (for determining the α_XY_ values), and pleasingly, this results in the correlation between HOMAc and NICS being noticeably better than the correlation between HOMA and NICS. Specifically, Figures 3 and S1 show that, for the eight quantum-chemical methods tested, the *R*^2^ values achieved by HOMAc are consistently 0.03–0.08 units larger than those attained by HOMA, with the corresponding average *R*^2^ values being 0.89 (HOMAc) and 0.84 (HOMA). As we will see, this improvement can indeed be traced to the better description of less aromatic and antiaromatic systems by HOMAc. Another positive feature of HOMAc is its robustness with respect to the choice of method for optimizing the geometries, with all associated *R*^2^ values in Figures 3 and S1 falling in a narrow 0.85–0.91 range and showing no bias against other methods than that used for the parametrization (CASPT2).

As for basis-set effects, some of the results in Figure 3 (the M06-2X ones) were also rederived by enlarging the basis set from cc-pVDZ to cc-pVTZ for all parts of the calculations. The updated results are shown in Figure S4 of the Supporting Information. Pleasingly, the cc-pVDZ and cc-pVTZ data are very similar in terms of the HOMAc-NICS and HOMA-NICS correlations they predict–in fact, the basis-set effect on the corresponding *R*^2^ values is no larger than 0.00 and 0.02 units, respectively. Furthermore, the two sets of data are also very similar at the level of the individual compounds–averaged over the 45 compounds, the basis-set effect is 0.02/0.04 for HOMAc/HOMA and 0.71 ppm for NICS_*zz*_(1).

**Figure 4:**
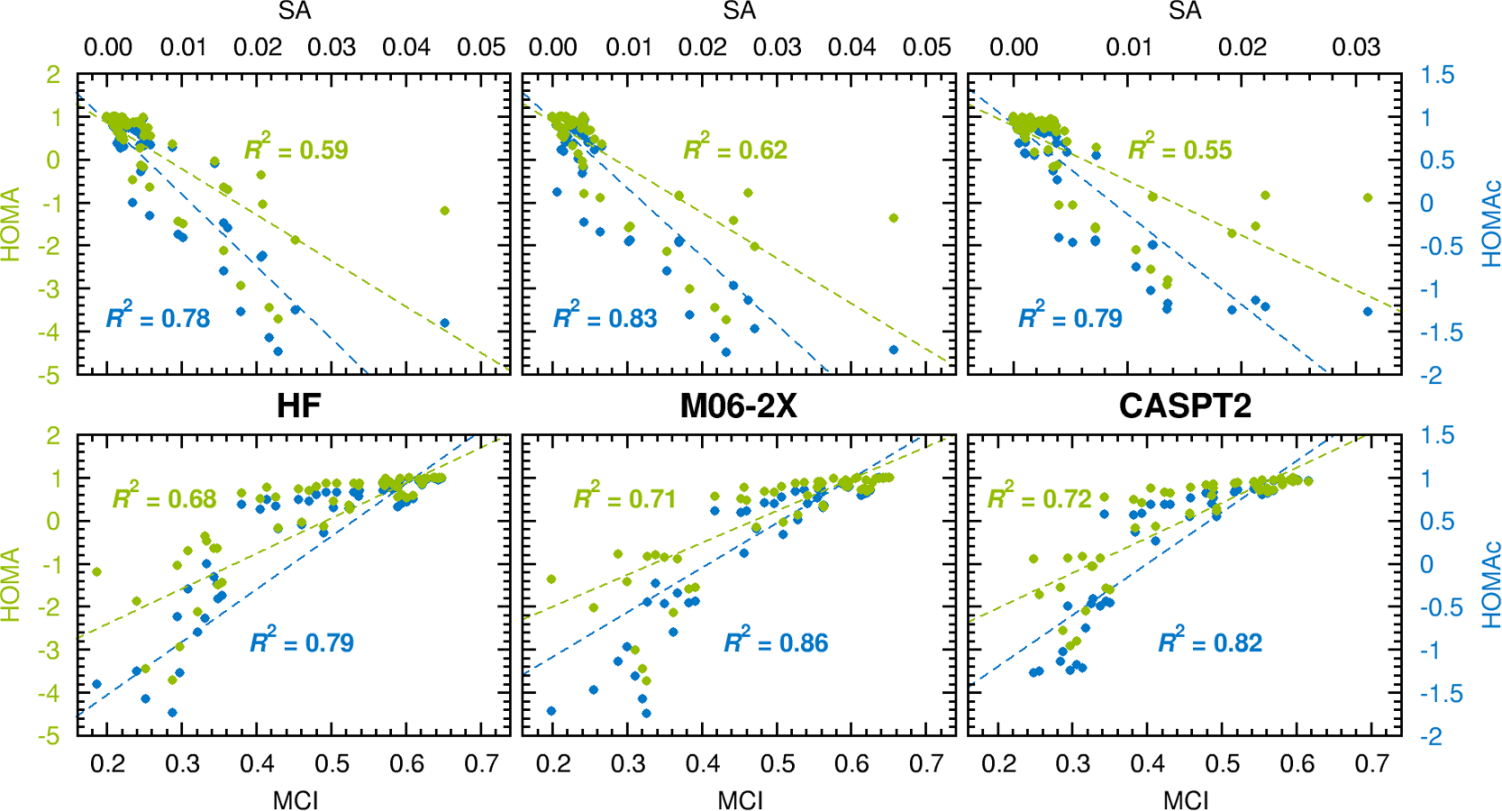
Linear correlations to SA (upper panels) and MCI values (lower panels) achieved by HOMAc (blue font) and HOMA values (green font) calculated at the HF, M06-2X and CASPT2 levels of theory.

As a further verification of the results in Figure 3, the calculated HOMAc and HOMA values were additionally compared with two other aromaticity indices than NICS that are also well-established but measure electron delocalization rather than induced ring currents. The indices in question are the MCI^11^ and Shannon aromaticity (SA)^77^ ones, both of which we calculated with the HF, M06-2X and CASSCF methods (that were also used to obtain the NICS data in Figure 3). The correlations with these indices that HOMAc and HOMA achieve are plotted in Figure 4. Here, it should be noted that aromaticity is associated with large MCI and small SA values, respectively.^11,77^ Interestingly, the results in Figure 4 corroborate the conclusion from Figure 3 that HOMAc offers a more reliable description of (anti)aromatic character than HOMA. In fact, in terms of *R*^2^ values, the improvement is even more pronounced when MCI (improvement by 0.10–0.15 units) and SA data (0.19–0.24) replace NICS data (0.03–0.08) as reference.

The trend that HOMAc performs better than HOMA is also reflected by the uncertainties (confidence bands) in how well these two indices reproduce the predictions by NICS, MCI and SA in Figures 3 and 4. Representing the uncertainties in terms of the confidence intervals for the linear-regression parameters (*y*-intercept and slope), the corresponding data are summarized in Table S4 of the Supporting Information. As can be seen, regardless of which combination of reference index (NICS, MCI or SA) and quantum-chemical method (HF, M06-2X or CASPT2) is considered, the confidence intervals attained by HOMAc are consistently narrower than those achieved by HOMA. In fact, the former are narrower by as much as 35–54%.

Next, the HOMAc and HOMA values for the monocyclic compounds in Figure 3 obtained with the M06-2X method were also compared with ISE values calculated with the same method. The ISE index is an energy-based measure of aromaticity and, consequently, provides reference data of a completely different origin than both NICS and MCI/SA. Specifically, this index quantifies the energy difference between a methylated derivative of the aromatic system in question and its nonaromatic, exocyclic methylene isomer.^8^ Accordingly, a strongly aromatic system is expected to show a distinctly negative ISE value, because the methylated species is then much more stable. Here, the ISE values were calculated using the isomeric pairs displayed in Figure S5 of the Supporting Information. Notably, the corresponding results, which are summarized in Figure S6 of the Supporting Information, provide yet further support for concluding that HOMAc (*R*^2^ value of 0.97 for the correlation with ISE) is a better aromaticity index than HOMA (0.88).

For reasons already outlined above, this work focuses on comparing HOMAc and HOMA in terms of the linear correlations they achieve with other aromaticity indices. However, it may also be of interest to explore nonlinear correlations, as a way to ensure that the conclusions on the relative merits of HOMAc and HOMA are not skewed by an intrinsic difference in how these two indices relate to the reference indices. To this end, the *R*^2^ values for the linear correlations shown in Figures 3 and 4 were compared with the *R*^2^ values obtained for the corresponding quadratic correlations. This comparison is made in Table S4. As expected from the functional forms, the quadratic *R*^2^ values are larger than the linear ones. Importantly, however, in each case considered, the trend from the linear correlations that the HOMAc *R*^2^ value is always larger than the HOMA *R*^2^ value, applies also to the quadratic correlations. For example, for the two possibilities, the magnitudes by which the *R*^2^ values for the HOMAc-NICS correlations exceed those for the HOMA-NICS correlations are 0.03–0.08 (linear) and 0.02–0.07 units (quadratic), respectively. Thus, our approach to focus on linear correlations does not bias the conclusions of this work.

### Importance of Including Antiaromaticity in the Parametrization

**Figure 5:**
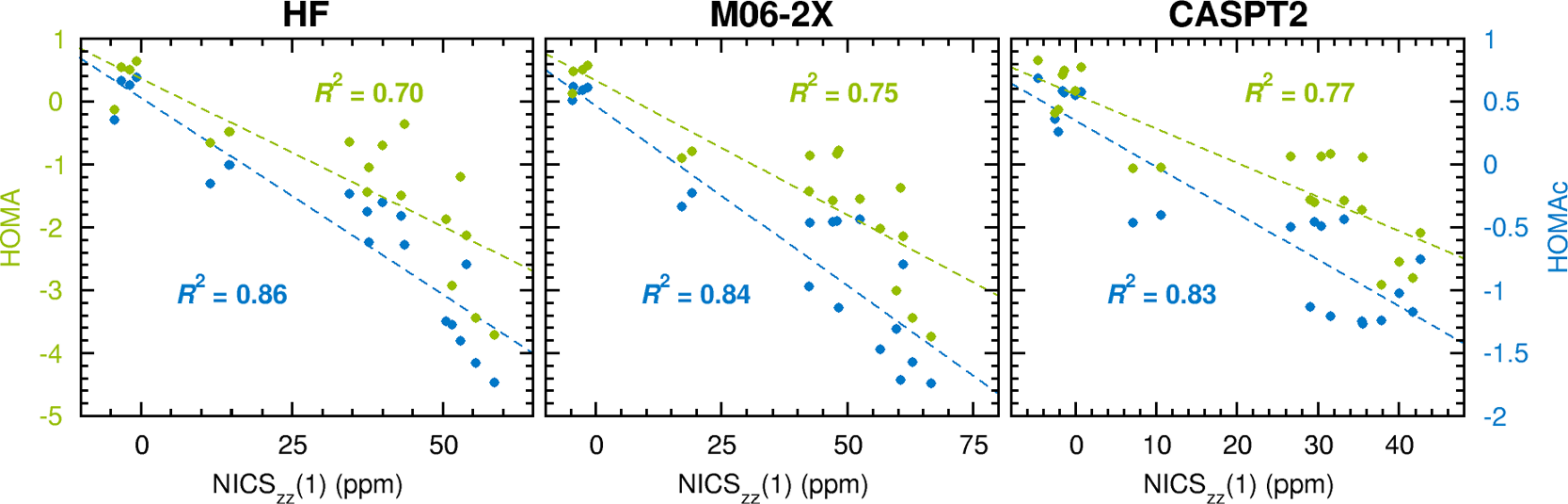
Linear correlations to NICS_*zz*_(1) values achieved by HOMAc (blue font) and HOMA values (green font) calculated at the HF, M06-2X and CASPT2 levels of theory, when the most distinctly aromatic systems are excluded from the analysis (here defined as those with NICS_*zz*_(1) values below −5 ppm).

Given that HOMAc includes antiaromaticity in the parametrization, and HOMA does not, the former’s improved performance over the latter may be the result of a better description of less aromatic and antiaromatic systems. In order to test this hypothesis, the analysis in Figure 3 was repeated, but now excluding the subset of the original data points/rings with NICS_*zz*_(1) values below –5 ppm. These data points, for which the HOMAc and HOMA values exceed 0.70, represent the most distinctly aromatic systems considered. Hence, by excluding these data points, the analysis of the remaining ones, which is presented in Figure 5, focuses on less aromatic and antiaromatic systems. Here, besides noting that the *R*^2^ values for HOMAc are larger than those for HOMA, it can also be seen that the corresponding margins are about twice as large (0.16, 0.09 and 0.06 units for HF, M06-2X and CASPT2, respectively) as they were for the original data set in Figure 3 (0.08, 0.04 and 0.03 units, respectively). Thus, the description of less aromatic and antiaromatic systems does indeed appear to be a key factor for the overall better performance of HOMAc.

With these results in hand, HOMAc and HOMA values were also calculated (using the M06-2X method) for two other categories of species than those included in the benchmark set of Figure 2: nonaromatic saturated compounds (cyclohexane, cyclobutane, cyclohexylamine, cyclobutylamine, cyclohexanecarbonitrile and cyclobutanecarbonitrile) and strongly antiaromatic transition-state geometries of cyclobutadiene (C_4_H_4_), aza-analogues of cyclobutadiene (C_2_N_2_H_2_ and N_4_) and cyclooctatetraene (C_8_H_8_). These results are given in Table S5 of the Supporting Information. As can be seen, neither HOMAc nor HOMA is consistent with the classifications of these species as nonaromatic and strongly antiaromatic, respectively. For the case of HOMAc, this may be due to an inherent inability of this parametrization, being based on the structures of conjugated potential-energy minima, to properly describe saturated potential-energy minima or conjugated transition states.

In connection to these results, it should be mentioned that regular HOMA indices perform the best when shorter bonds are stronger (in terms of force constants) than longer bonds,^13^ as prescribed by Badger’s rule.^78^ However, there are certainly exceptions to this rule.^79,80^ In such situations, a HOMA-like index formulated in terms of stretching force constants obtained from local vibrational mode theory,^81,82^ rather than in terms of bond lengths, has been found to improve the description of aromaticity and antiaromaticity.^13^

### Separate Comparisons of HOMAc and HOMA for Monocyclic and Polycyclic Systems

**Figure 6:**
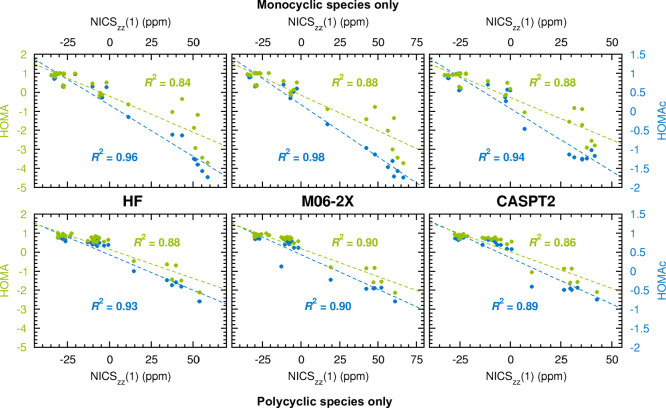
Linear correlations to NICS_*zz*_(1) values achieved by HOMAc (blue font) and HOMA values (green font) calculated at the HF, M06-2X and CASPT2 levels of theory, when the benchmark set of investigated compounds is divided into two subsets containing exclusively monocyclic (upper panels) or polycyclic (lower panels) species.

Since the HOMAc parametrization derives entirely from calculated bond lengths of monocyclic compounds (see Figure 1), which are frequent among the 45 compounds constituting the benchmark set (see Figure 2), it is also of interest to compare the performances of HOMAc and HOMA for monocyclic and polycyclic systems separately. Accordingly, the benchmark set was divided into one subset containing compounds **1**–**28** (exclusively monocyclic species) and another subset containing compounds **29**–**45** (exclusively polycyclic species). Then, the degrees to which calculated HOMAc and HOMA values correlate with NICS_*zz*_(1) values were compared for the two subsets. These comparisons are presented in Figure 6 (for the HF, M06-2X and CASPT2 methods) and in Figures S7 (M06-L, B3LYP and ωB97X-D) and S8 (MP2 and CCSD) of the Supporting Information. For the polycyclic species, all of which feature two fused rings, the procedure to assign local (anti)aromatic character to each ring based on calculated NICS and HOMA values follows a long tradition in studies of aromatic compounds of this type.^83−92^

Starting with the monocyclic systems, an analysis of the results obtained by all eight methods in Figures 6, S7, and S8 shows that the HOMAc *R*^2^ values are consistently 0.06–0.12 units larger than the HOMA ones. With the average *R*^2^ values being 0.95 (HOMAc) and 0.86 (HOMA), the corresponding 0.09-unit improvement achieved by HOMAc is greater than that documented above for the full benchmark set (0.05 units, see Figures 3 and S1). Thus, the better performance of HOMAc is accentuated for monocyclic systems. However, despite that the parametrization is based exclusively on monocyclic compounds, HOMAc improves upon HOMA also for the polycyclic systems, albeit by a lesser margin. Specifically, for these systems, the *R*^2^ values for HOMAc are consistently 0.00–0.05 units larger (see Figures 6, S7, and S8), with the average *R*^2^ value for HOMAc (0.92) exceeding that for HOMA (0.88) by 0.04 units. This ability of HOMAc to perform well also for systems beyond the immediate range of the parametrization is a clear positive sign for the future usage of this model as a simple but reliable tool in detecting and quantifying aromaticity and antiaromaticity.

Finally, as for possible avenues for further development and testing of HOMAc, one natural goal would be to expand the parametrization to include a wider range of bonds than considered in this study. Given the importance of aromaticity for thiophene chemistry, CS bonds are here of particular interest. Another worthwhile task would be to compare HOMAc and HOMA for more specific applications, such as describing substituents effects on the aromaticity of pharmaceutically relevant motifs like five-membered N-heterocycles,^93,94^ or describing position isomers of certain heterocycles for which aromaticity and stability are not necessarily correlated.^95^ Moreover, it would also be of interest to explore the relationships of HOMAc and HOMA with Clar’s rule^96,97^ for identifying the most important resonance structures of polycyclic aromatic hydrocarbons and, given the inclusion of antiaromaticity in the parametrization of HOMAc, test whether HOMAc performs better than HOMA in identifying and quantifying local antiaromaticity in such compounds. For the latter task, suitable magnetic reference data have recently been reported.^98^

## Conclusions

In summary, we have presented a new parametrization of the HOMA index that includes antiaromaticity and where the *R*_opt_ and α parameters are derived from the actual bond lengths of model aromatic and antiaromatic compounds calculated with a high-level ab initio method (CASPT2). Focusing on CC, CN, NN and CO bonds, the resulting parametrization is termed HOMAc (where “c” stands for computational) and is subjected to extensive testing comparing its performance in probing (anti)aromatic character to that of the standard 1993 parametrization of HOMA,^22^ using eight different quantum-chemical methods (four ab initio and four DFT). Employing a benchmark set of 45 molecules across the aromatic-antiaromatic spectrum, including both single-ring and multi-ring carbocyclic and N,O-heterocyclic systems, these tests show that, at each quantum-chemical level considered, HOMAc yields a more reliable description of (anti)aromaticity than HOMA. Specifically, this can be inferred from the better linear correlations that HOMAc achieves with four other, commonly used aromaticity indices in the form of NICS (improvement over HOMA by 0.03–0.08 *R*^2^ units), MCI (0.10–0.15), SA (0.19–0.24) and ISE (0.09).

Altogether, varying the reference index and the quantum-chemical method and considering different parts of the benchmark set, this work compares HOMAc and HOMA based on 41 (linear) + 9 (quadratic) = 50 *R*^2^ values. In 49 of these cases, the *R*^2^ value for HOMAc is larger than the *R*^2^ value for HOMA (in the remaining case, the two *R*^2^ values are equal). In more specific terms, the *R*^2^ value for HOMAc is on average 0.08 units larger for linear correlations, and 0.10 units larger for quadratic correlations. In this light, the conclusion that HOMAc performs better than HOMA is clearly well-founded.

By assessing the changes in the margins by which the HOMAc-NICS correlations exceed the HOMA-NICS correlations when distinctly aromatic systems are excluded from the benchmark set, it is furthermore concluded that a particular key factor for the better performance of HOMAc is the description of less aromatic and antiaromatic systems, which is consistent with the inclusion of antiaromaticity in the parametrization. As for separate comparisons of HOMAc and HOMA for monocyclic and polycyclic molecules, the improvement accomplished by HOMAc is greater for the former molecules, in accord with our strategy to only include such compounds in the parametrization. Importantly, however, HOMAc performs better than HOMA also for the polycyclic compounds. Thus, for the types of bonds that we have considered (CC, CN, NN and CO), the overall picture emerging from this work is that HOMAc is a more accurate aromaticity index than HOMA. Combined with the observation that the HOMAc predictions are quite equally correlated with NICS data at all levels of theory tested, this suggests that HOMAc can find broad applicability in future studies of aromaticity and antiaromaticity.

## Computational Methods

All calculations except where otherwise noted were carried out with the cc-pVDZ basis set.^58^ All CASSCF and CASPT2 calculations were performed with active spaces including only π and π* orbitals. This means (4,4) and (6,6) active spaces for the HOMAc parametrization compounds in Figure 1 with four- and six-membered rings, respectively, and active spaces ranging from (4,4) to (12,10) for the benchmark compounds in Figure 2 (see also Table S6 of the Supporting Information).

The CASPT2 geometry optimizations were performed with the BAGEL 1.1.2 software.^99^ The optimizations for the HOMAc parametrization were done with the larger cc-pVQZ basis set^58^ and tighter convergence criteria (0.00001 hartree Bohr^–1^ for the maximum component of the gradient vector, 0.00004 Bohr for the maximum component of the displacement vector, and 0.000001 hartree for the maximum energy change) than the default ones used by BAGEL 1.1.2. All other CASPT2 optimizations were done with the default convergence criteria. The HF, MP2, CCSD, M06-L, M06-2X, B3LYP and ωB97X-D geometry optimizations were carried out with the Gaussian 16 software.^100^ All structures produced by the geometry optimizations were characterized by frequency calculations. For any given structure, the corresponding calculation was done at the same level of theory and with the same software used to optimize that structure. At the CASPT2 and CCSD levels, the frequency calculations were performed numerically, and at all other levels, they were performed analytically. From the frequency calculations, all optimized geometries were found to be potential-energy minima with real vibrational frequencies only.

As for the calculation of aromaticity indices, the HOMA and HOMAc values for the different sets of optimized geometries were obtained with the Multiwfn 3.7 software.^101^ Specifically, standard (with the 1993 HOMA parameters^22^) and modified (with the HOMAc parameters derived in this work) versions of this software were used to obtain HOMA and HOMAc values, respectively. Multiwfn 3.7 was also employed to calculate the reported SA and MCI values (based on electron densities optimized with the HF, M06-2X and CASSCF methods). The NICS_*zz*_ values, finally, were calculated with Gaussian 16 (HF, MP2, M06-L, M06-2X, B3LYP and ωB97X-D) and the Dalton 2016.2 software^102,103^ (CASSCF), using gauge-including atomic orbitals in each case.

Numerical integration in all DFT calculations was carried out with the default grid size in Gaussian 16 (“Ultrafine”) comprising 99 radial shells and 590 angular points per shell.

## Data Availability

The data underlying this study are available in the published article and its Supporting Information.

## Supporting Information Available

Cartesian coordinates and energies for the parametrization compounds in Figure 1; Cartesian coordinates, energies, and aromaticity indices for the benchmark compounds in Figure 2; complementary correlations between aromaticity indices; calculated aromaticity indices for other species; and active spaces in CASSCF and CASPT2 calculations (PDF)
