# Supplementary material for: HOMAc: A Parameterization of the Harmonic Oscillator Model of Aromaticity (HOMA) That Includes Antiaromaticity
Source: J Org Chem. 2025 Jan 15;90(3):1297–308. doi: 10.1021/acs.joc.4c02475 (PMC11773415; doi:10.1021/acs.joc.4c02475)
Supplement: Supplementary file 1 — jo4c02475_si_001.pdf [file jo4c02475_si_001.pdf]

HOMAc: A Parameterization of the Harmonic Oscillator Model of Aromaticity (HOMA) that Includes Antiaromaticity

Enrique M. Arpa,*†‡ Sven Stafström,§ and Bo Durbeej*†

†Division of Theoretical Chemistry, IFM, Linköping University, 58183 Linköping, Sweden
‡Institute of Organic Chemistry, RWTH Aachen University, 52056 Aachen, Germany
§Division of Theoretical Physics, IFM, Linköping University, 58183 Linköping, Sweden

Supporting Information

Table of Contents

1. CASPT2/cc-pVQZ Cartesian Coordinates and Energies for the Parameterization Compounds in Figure 1 — page S2
2. HF/cc-pVDZ Cartesian Coordinates, Energies and Aromaticity Indices (Table S1) for the Benchmark Compounds in Figure 2 — page S4
3. M06-2X/cc-pVDZ Cartesian Coordinates, Energies and Aromaticity Indices (Table S2) for the Benchmark Compounds in Figure 2 — page S18
4. CASPT2/cc-pVDZ Cartesian Coordinates, Energies and Aromaticity Indices (Table S3) for the Benchmark Compounds in Figure 2 — page S32
5. Complementary Correlations Between Aromaticity Indices (Figures S1–S8) — page S46
6. Comparison of Linear and Quadratic Correlations Between Aromaticity Indices (Table S4) — page S51
7. Calculated Aromaticity Indices for Other Species (Table S5) — page S52
8. Active Spaces in CASSCF and CASPT2 Calculations (Table S6) — page S53

Corresponding Authors
*Email: enrique.arpa@rwth-aachen.de (E.M.A.)
*Email: bodur@ifm.liu.se (B.D.)

1. CASPT2/cc-pVQZ Cartesian Coordinates and Energies for the Parameterization Compounds in Figure 1

Cartesian coordinates in Å and electronic energies (E) in Hartree atomic units (a.u.).

C_6H_6 (used to obtain $R_{CC,opt}$)
E = -231.79478938 a.u. (all vibrational frequencies real)

C	-0.000000	1.391905	0.000000
C	-1.205425	0.695952	0.000000
C	-1.205425	-0.695952	-0.000000
C	0.000000	-1.391905	-0.000000
C	1.205425	-0.695952	-0.000000
C	1.205425	0.695953	0.000000
H	0.000000	2.471257	0.000000
H	-2.140172	1.235629	-0.000000
H	-2.140172	-1.235629	-0.000000
H	0.000000	-2.471257	0.000000
H	2.140172	-1.235629	0.000000
H	2.140172	1.235629	0.000000

C_4H_4 (used to obtain α_{CC})
E = -154.38090098 a.u. (all vibrational frequencies real)

C	0.780166	0.668452	0.000000
C	0.767915	-0.682493	0.000000
C	-0.780166	-0.668452	0.000000
C	-0.767915	0.682493	-0.000000
H	1.547477	1.422501	-0.000000
H	1.521423	-1.450335	-0.000000
H	-1.547477	-1.422501	0.000000
H	-1.521423	1.450335	-0.000000

$C_3N_3H_3$ (used to obtain $R_{CN,opt}$)
E = -279.90812637 a.u. (all vibrational frequencies real)

N	-0.000000	1.371406	0.000000
C	1.117362	0.645109	0.000000
N	1.187673	-0.685703	0.000000
C	-0.000000	-1.290218	0.000000
N	-1.187673	-0.685703	-0.000000
C	-1.117362	0.645109	-0.000000
H	2.052998	1.185299	-0.000000
H	-0.000000	-2.370598	0.000000
H	-2.052998	1.185299	0.000000

$C_2N_2H_2$ (used to obtain α_{CN})
E = -186.47215131 a.u. (all vibrational frequencies real)

C	0.747750	0.512040	0.000000
N	0.745448	-0.774468	0.000000
C	-0.747750	-0.512040	0.000000
N	-0.745448	0.774468	-0.000000
H	1.559435	1.226834	-0.000000
H	-1.559435	-1.226834	0.000000

N_6 (used to obtain $R_{NN,opt}$)
E = -327.81884852 a.u. (all vibrational frequencies real)

N	0.000000	1.317845	0.000000
N	-1.141287	0.658922	0.000000
N	-1.141287	-0.658922	-0.000000

N	0.000000	-1.317845	-0.000000
N	1.141287	-0.658923	0.000000
N	1.141287	0.658923	0.000000

N_4 (used to obtain α_{NN})
E = -218.47051587 a.u. (all vibrational frequencies real)

N	0.627592	-0.761859	0.000000
N	-0.640802	-0.750780	0.000000
N	-0.627592	0.761859	0.000000
N	0.640802	0.750780	-0.000000

$C_3O_3H_3$ (used to obtain $R_{CO,opt}$)
E = -339.59149001 a.u. (all vibrational frequencies real)

O	-0.000000	1.304933	0.000000
C	1.148113	0.662863	0.000000
O	1.130105	-0.652467	-0.000000
C	-0.000000	-1.325726	-0.000000
O	-1.130105	-0.652467	-0.000000
C	-1.148113	0.662863	0.000000
H	2.113265	1.220094	0.000000
H	-0.000000	-2.440188	-0.000000
H	-2.113265	1.220094	0.000000

$C_2O_2H_2$ (used to obtain α_{CO})
E = -226.48060136 a.u. (all vibrational frequencies real)

C	0.690629	0.581474	0.000000
O	0.724978	-0.705421	0.000000
C	-0.690629	-0.581474	0.000000
O	-0.724978	0.705421	-0.000000
H	1.467991	1.371101	0.000000
H	-1.467991	-1.371101	0.000000

2. HF/cc-pVDZ Cartesian Coordinates, Energies and Aromaticity Indices (Table S1) for the Benchmark Compounds in Figure 2

Cartesian coordinates in Å and electronic energies (*E*) in Hartree atomic units (a.u.).

Compound **1**
E = -230.722349565 a.u. (all vibrational frequencies real)

C	0.000000	1.388667	-0.000000
C	-1.202621	0.694334	0.000000
C	-1.202621	-0.694334	0.000000
C	0.000000	-1.388667	-0.000000
C	1.202621	-0.694334	-0.000000
C	1.202621	0.694334	-0.000000
H	0.000000	2.470823	-0.000000
H	-2.139795	1.235411	0.000000
H	-2.139795	-1.235411	0.000000
H	0.000000	-2.470823	-0.000000
H	2.139795	-1.235411	-0.000000
H	2.139795	1.235411	-0.000000

Compound **2**
E = -246.715726544 a.u. (all vibrational frequencies real)

N	-1.185448	0.753347	-0.000000
C	0.000000	1.340141	0.000000
C	1.202398	0.647721	0.000000
C	1.161034	-0.737839	0.000000
C	-0.075823	-1.363654	-0.000000
C	-1.213314	-0.569076	-0.000000
H	-0.003852	2.423545	-0.000000
H	2.140727	1.184134	0.000000
H	2.074341	-1.318243	0.000000
H	-0.163028	-2.440963	-0.000000
H	-2.195818	-1.025661	-0.000000

Compound **3**
E = -262.671751694 a.u. (all vibrational frequencies real)

N	-1.172157	-0.723341	-0.000000
N	-1.248505	0.581726	0.000000
C	-0.153072	1.303793	0.000000
C	1.129153	0.752378	0.000000
C	1.209180	-0.615580	-0.000000
C	0.000000	-1.312747	-0.000000
H	-0.302584	2.375269	0.000000
H	2.005675	1.385269	0.000000
H	2.153524	-1.141943	-0.000000
H	-0.023554	-2.394348	-0.000000

Compound **4**
E = -262.714678696 a.u. (all vibrational frequencies real)

N	1.182508	0.708735	0.000000
C	0.000000	1.296380	0.000000
N	-1.182508	0.708735	0.000000
C	-1.174058	-0.613370	0.000000
C	-0.000000	-1.347298	0.000000
C	1.174057	-0.613370	0.000000
H	0.000000	2.378916	0.000000
H	-2.140321	-1.104044	0.000000

H	-0.000000	-2.427176	0.000000
H	2.140321	-1.104045	0.000000

Compound **5**

E = -262.704184623 a.u. (all vibrational frequencies real)

N	0.000000	0.000000	1.390278
C	0.000000	1.121935	0.693908
C	0.000000	1.121935	-0.693908
N	0.000000	0.000000	-1.390278
C	-0.000000	-1.121935	-0.693908
C	-0.000000	-1.121935	0.693908
H	0.000000	2.051929	1.247283
H	0.000000	2.051929	-1.247283
H	-0.000000	-2.051929	-1.247283
H	-0.000000	-2.051929	1.247283

Compound **6**

E = -278.718814691 a.u. (all vibrational frequencies real)

N	-1.174873	0.678318	-0.000000
C	0.000000	1.279129	0.000000
N	1.174877	0.678311	0.000000
C	1.107758	-0.639564	0.000000
N	-0.000004	-1.356629	-0.000000
C	-1.107758	-0.639564	-0.000000
H	0.000005	2.361987	0.000000
H	2.045538	-1.180998	0.000000
H	-2.045543	-1.180989	-0.000000

Compound **7**

E = -321.595636959 a.u. (all vibrational frequencies real)

N	-1.129918	0.257987	0.000000
C	0.000000	1.057804	0.000000
C	1.250176	0.305146	0.000000
C	1.253004	-1.040390	-0.000000
C	0.026673	-1.794731	-0.000000
C	-1.124928	-1.104128	-0.000000
H	2.159661	0.886795	0.000000
H	2.195777	-1.573185	-0.000000
H	0.026683	-2.872969	-0.000000
H	-2.096034	-1.577759	-0.000000
O	-0.101089	2.254192	0.000000
H	-1.997495	0.755471	0.000000

Compound **8**

E = -321.597849159 a.u. (all vibrational frequencies real)

N	-1.167707	0.306094	-0.000000
C	-0.000000	0.898683	-0.000000
C	1.222347	0.219546	0.000000
C	1.180167	-1.154970	0.000000
C	-0.054510	-1.808328	0.000000
C	-1.187229	-1.024749	-0.000000
H	2.147110	0.776095	0.000000
H	2.100957	-1.724030	0.000000
H	-0.126053	-2.885935	0.000000
H	-2.170337	-1.479202	-0.000000
O	0.017608	2.232290	-0.000000
H	-0.883242	2.531001	-0.000000

Compound **9**

E = -412.508486137 a.u. (all vibrational frequencies real)

N	-1.136489	-1.012864	-0.000000
C	-1.218524	0.359707	-0.000000
N	-0.000000	0.985650	0.000000
C	1.261037	0.396435	0.000000
C	1.231544	-1.067221	0.000000
C	0.054434	-1.692409	0.000000
H	2.167747	-1.599343	0.000000
H	-0.029216	-2.769554	-0.000000
O	-2.258631	0.940910	-0.000000
O	2.243796	1.070326	0.000000
H	-2.009304	-1.496609	-0.000000
H	-0.026056	1.987037	-0.000000

Compound **10**
E = -412.488754370 a.u. (all vibrational frequencies real)

N	1.194605	-1.050484	0.000000
C	1.102563	0.261202	0.000000
N	0.000000	0.990184	0.000000
C	-1.128467	0.321138	0.000000
C	-1.188185	-1.075891	0.000000
C	0.033434	-1.701401	0.000000
H	-2.127597	-1.603310	0.000000
H	0.097124	-2.782730	0.000000
O	2.229457	0.947817	0.000000
O	-2.250488	1.016426	0.000000
H	2.944886	0.324454	0.000000
H	-2.024471	1.939465	0.000000

Compound **11**
E = -341.405458244 a.u. (all vibrational frequencies real)

O	0.329320	-1.137318	0.000048
C	1.045425	0.024690	0.000400
C	0.252493	1.254440	0.000030
C	-1.084302	1.205506	0.000036
C	-1.764503	-0.073393	0.000066
C	-1.007657	-1.167962	-0.000016
O	2.224584	-0.041235	-0.000394
H	0.811685	2.177202	-0.000155
H	-1.665625	2.118353	-0.000067
H	-2.840074	-0.148261	0.000021
H	-1.385945	-2.178554	-0.000126

Compound **12**
E = -341.392057007 a.u. (all vibrational frequencies real)

O	1.736564	-0.000000	0.000022
C	1.046416	1.151136	-0.000051
C	-0.281591	1.224429	-0.000074
C	-1.096236	-0.000000	-0.000029
C	-0.281591	-1.224429	0.000066
C	1.046416	-1.151136	0.000080
H	1.705821	2.004542	-0.000087
H	-0.774502	2.184288	-0.000129
O	-2.294454	0.000000	-0.000021
H	-0.774501	-2.184288	0.000119
H	1.705821	-2.004542	0.000141

Compound **13**
E = -285.758650762 a.u. (all vibrational frequencies real)

C	-0.933398	0.000000	-0.014046
C	-0.220681	-1.199820	-0.008162

C	1.165149	-1.194084	0.004188
C	1.872819	0.000001	0.011168
C	1.165149	1.194084	0.004189
C	-0.220682	1.199819	-0.008158
H	-0.757764	-2.140138	-0.018946
H	1.696481	-2.137207	0.009406
H	2.953854	0.000001	0.021390
H	1.696480	2.137208	0.009407
H	-0.757764	2.140138	-0.018936
N	-2.329193	-0.000004	-0.080416
H	-2.748536	-0.819936	0.312796
H	-2.748537	0.819963	0.312722

Compound **14**
E = -322.463815902 a.u. (all vibrational frequencies real)

C	0.000000	0.600609	0.000000
C	1.208624	-0.091127	-0.000000
C	1.204324	-1.476698	-0.000000
C	-0.000187	-2.168000	-0.000000
C	-1.204619	-1.476513	-0.000000
C	-1.208736	-0.090962	-0.000000
H	2.140051	0.456520	0.000000
H	2.140886	-2.016623	-0.000000
H	-0.000265	-3.249644	-0.000000
H	-2.141253	-2.016313	-0.000000
H	-2.140073	0.456839	0.000000
C	0.000014	2.047684	0.000000
N	0.000591	3.185609	0.000000

Compound **15**
E = -153.656403041 a.u. (all vibrational frequencies real)

C	-0.784041	-0.661558	0.000000
C	-0.784041	0.661558	0.000000
C	0.784041	0.661558	0.000000
C	0.784041	-0.661558	0.000000
H	-1.546526	-1.425862	0.000000
H	-1.546527	1.425861	0.000000
H	1.546526	1.425862	0.000000
H	1.546527	-1.425861	0.000000

Compound **16**
E = -169.659645374 a.u. (all vibrational frequencies real)

N	-1.036049	0.235888	0.000000
C	-0.000000	0.956700	0.000000
C	1.006292	-0.220239	0.000000
C	-0.091731	-0.955382	0.000000
H	0.056442	2.041064	0.000000
H	2.072530	-0.378112	0.000000
H	-0.363993	-2.000639	0.000000

Compound **17**
E = -185.623079466 a.u. (all vibrational frequencies real)

N	-0.997343	-0.178380	0.000000
N	-0.101085	-1.008097	0.000000
C	0.966030	0.074569	0.000000
C	-0.000000	0.968902	0.000000
H	2.042646	0.007357	0.000000
H	-0.149831	2.037155	0.000000

Compound **18**

E = -185.671571216 a.u. (all vibrational frequencies real)

C	0.734288	0.506239	0.000000
N	0.734288	-0.758289	0.000000
C	-0.734288	-0.506239	0.000000
N	-0.734288	0.758289	0.000000
H	1.550803	1.221959	0.000000
H	-1.550803	-1.221959	0.000000

Compound **19**

E = -244.606287034 a.u. (all vibrational frequencies real)

N	-0.951753	-0.455401	0.000000
C	-0.000000	0.593988	0.000000
C	1.096229	-0.462476	0.000000
C	0.122697	-1.375428	0.000000
H	2.169899	-0.460529	0.000000
H	0.050433	-2.451763	0.000000
O	-0.115839	1.760068	0.000000
H	-1.944909	-0.516953	0.000000

Compound **20**

E = -244.555036201 a.u. (all vibrational frequencies real)

N	-1.066918	-0.279327	-0.000000
C	-0.000000	0.420146	-0.000000
C	0.998184	-0.727007	0.000000
C	-0.122771	-1.439628	0.000000
H	2.063615	-0.871044	0.000000
H	-0.399046	-2.483955	0.000000
O	0.156914	1.708867	-0.000000
H	-0.703933	2.118285	-0.000000

Compound **21**

E = -208.703093091 a.u. (all vibrational frequencies real)

C	-0.452349	0.058884	0.002204
C	0.420503	1.075191	0.019009
C	1.556364	0.018672	0.002976
C	0.695935	-0.991642	-0.001384
H	0.369525	2.149687	-0.007669
H	2.636127	0.075254	-0.006961
H	0.748854	-2.067755	-0.028868
N	-1.796588	-0.121029	-0.080056
H	-2.143684	-0.959287	0.341868
H	-2.357431	0.682667	0.125193

Compound **22**

E = -245.401540602 a.u. (all vibrational frequencies real)

C	-0.000000	0.100746	-0.000000
C	1.311164	-0.099999	0.000000
C	1.081597	-1.638984	0.000000
C	-0.228035	-1.451599	0.000000
H	2.172699	0.550322	0.000000
H	1.734382	-2.496424	0.000000
H	-1.103653	-2.081324	0.000000
C	-0.841239	1.249223	-0.000000
N	-1.534907	2.153015	-0.000000

Compound **23**

E = -208.829121064 a.u. (all vibrational frequencies real)

C	0.000000	1.113867	0.328095
C	0.000000	0.714539	-0.974371
C	-0.000000	-0.714539	-0.974371

C	-0.000000	-1.113867	0.328095
N	-0.000000	0.000000	1.113995
H	-0.000000	0.000000	2.109174
H	0.000000	2.101175	0.758586
H	0.000000	1.364096	-1.834503
H	-0.000000	-1.364096	-1.834503
H	-0.000000	-2.101175	0.758586

Compound **24**

E = -224.815759512 a.u. (all vibrational frequencies real)

N	-0.000000	1.075279	0.000000
N	-1.126459	0.373971	0.000000
C	-0.749278	-0.874081	0.000000
C	0.662352	-0.993344	0.000000
C	1.098015	0.302345	0.000000
H	-0.041697	2.070370	0.000000
H	-1.487460	-1.660135	0.000000
H	1.258857	-1.888957	0.000000
H	2.088980	0.724448	0.000000

Compound **25**

E = -224.836354813 a.u. (all vibrational frequencies real)

N	0.000000	1.096156	0.000000
C	-1.075556	0.279612	0.000000
N	-0.742247	-0.968281	0.000000
C	0.629987	-0.976067	0.000000
C	1.111353	0.290825	0.000000
H	-0.014841	2.092063	0.000000
H	-2.085157	0.658542	0.000000
H	1.188870	-1.896892	0.000000
H	2.112152	0.684952	0.000000

Compound **26**

E = -228.644520616 a.u. (all vibrational frequencies real)

O	0.000000	1.142963	0.000101
C	-1.081375	0.346170	0.000023
C	-0.721950	-0.948286	-0.000105
C	0.721950	-0.948286	0.000056
C	1.081375	0.346170	-0.000091
H	-2.037006	0.839526	0.000069
H	-1.382885	-1.798682	-0.000169
H	1.382885	-1.798682	0.000108
H	2.037006	0.839526	-0.000118

Compound **27**

E = -244.653496893 a.u. (all vibrational frequencies real)

O	-0.337330	-1.071725	0.000016
C	-1.085030	0.026714	-0.000085
N	-0.436817	1.119784	-0.000091
C	0.893473	0.718809	0.000037
C	0.938419	-0.617980	0.000114
H	-2.153143	-0.106216	-0.000171
H	1.704221	1.425904	0.000066
H	1.724104	-1.349643	0.000216

Compound **28**

E = -343.419363344 a.u. (all vibrational frequencies real)

C	-0.000000	-0.000000	1.323895
C	0.000000	1.293406	0.610496
C	0.000000	1.564063	-0.700870

C	0.000000	0.669961	-1.843417
C	-0.000000	-0.669961	-1.843417
C	-0.000000	-1.564063	-0.700870
C	-0.000000	-1.293406	0.610496
O	-0.000000	-0.000000	2.525352
H	0.000000	2.127829	1.299990
H	0.000000	2.615843	-0.962324
H	0.000000	1.161738	-2.808011
H	-0.000000	-1.161738	-2.808011
H	-0.000000	-2.615843	-0.962324
H	-0.000000	-2.127829	1.299990

Compound **29**

E = -383.384958890 a.u. (all vibrational frequencies real)

C	1.244167	-1.394891	-0.000000
C	2.420168	-0.709279	-0.000000
C	2.420168	0.709279	-0.000000
C	1.244167	1.394891	0.000000
C	0.000000	0.705749	0.000000
C	0.000000	-0.705749	-0.000000
C	-1.244168	-1.394891	-0.000000
C	-2.420168	-0.709279	0.000000
C	-2.420168	0.709279	0.000000
C	-1.244168	1.394891	0.000000
H	1.242463	-2.477428	-0.000000
H	3.361097	-1.243435	-0.000000
H	3.361097	1.243435	-0.000000
H	1.242463	2.477428	0.000000
H	-1.242463	-2.477428	-0.000000
H	-3.361097	-1.243435	0.000000
H	-3.361097	1.243435	0.000000
H	-1.242463	2.477428	0.000000

Compound **30**

E = -399.380655577 a.u. (all vibrational frequencies real)

C	1.264894	1.340334	0.000000
C	2.412745	0.608710	0.000000
C	2.364919	-0.810108	-0.000000
C	1.165895	-1.453725	-0.000000
C	-0.047940	-0.714380	-0.000000
C	-0.000000	0.693014	0.000000
N	-1.118594	1.462719	0.000000
C	-2.267998	0.869133	0.000000
C	-2.437859	-0.540325	-0.000000
C	-1.328703	-1.325460	-0.000000
H	1.277399	2.420718	0.000000
H	3.373000	1.107247	0.000000
H	3.287204	-1.375344	-0.000000
H	1.121592	-2.535291	-0.000000
H	-3.144377	1.508095	0.000000
H	-3.432989	-0.962329	-0.000000
H	-1.407381	-2.405296	-0.000000

Compound **31**

E = -399.378684697 a.u. (all vibrational frequencies real)

C	1.241861	1.374836	0.000000
C	2.411095	0.677618	0.000000
C	2.391915	-0.742026	0.000000
C	1.213586	-1.424905	0.000000
C	-0.020068	-0.718817	-0.000000

C	0.000000	0.686723	-0.000000
C	-1.251359	1.364930	-0.000000
N	-2.401698	0.774990	-0.000000
C	-2.413068	-0.580689	-0.000000
C	-1.291952	-1.350132	-0.000000
H	1.245724	2.457317	0.000000
H	3.359027	1.198290	0.000000
H	3.328905	-1.283476	0.000000
H	1.204172	-2.506868	0.000000
H	-1.256969	2.450328	-0.000000
H	-3.394347	-1.036701	-0.000000
H	-1.366686	-2.429054	-0.000000

Compound **32**

E = -409.506073333 a.u. (all vibrational frequencies real)

N	-1.257186	1.081986	0.000000
C	-2.101991	0.067343	0.000000
N	-1.819445	-1.231157	0.000000
C	-0.547102	-1.592508	0.000000
C	0.444983	-0.628727	-0.000000
C	-0.000000	0.691409	0.000000
N	1.134753	1.447259	-0.000000
C	2.182427	0.560753	-0.000000
N	1.830160	-0.671166	-0.000000
H	-3.153339	0.322193	0.000000
H	-0.323468	-2.651920	-0.000000
H	1.185338	2.443061	-0.000000
H	3.203595	0.908582	-0.000000

Compound **33**

E = -464.563184449 a.u. (all vibrational frequencies real)

N	-0.012652	1.902085	0.000599
C	1.282993	1.678439	0.004670
N	1.930533	0.516299	0.003696
C	1.218630	-0.603489	-0.008719
C	-0.179547	-0.511150	-0.006537
C	-0.701574	0.765421	-0.002610
N	-2.052849	0.588833	0.001731
C	-2.267607	-0.766615	0.004122
N	-1.188688	-1.459255	-0.000431
H	1.916284	2.556581	0.011610
H	-2.741614	1.309501	0.004818
H	-3.265957	-1.174126	0.009350
N	1.865227	-1.788360	-0.052167
H	2.836903	-1.776113	0.176060
H	1.346026	-2.608699	0.178607

Compound **34**

E = -484.392785782 a.u. (all vibrational frequencies real)

N	-0.598601	-1.770560	-0.000000
C	0.659469	-1.998044	0.000000
N	1.625045	-1.048626	0.000000
C	1.418545	0.346990	0.000000
C	0.000000	0.617122	0.000000
C	-0.875513	-0.435818	-0.000000
N	-2.110832	0.121231	-0.000000
C	-1.921016	1.478061	-0.000000
N	-0.681472	1.811806	-0.000000
H	1.012423	-3.021242	0.000000
H	-2.977423	-0.372239	-0.000000

H	-2.752423	2.163811	-0.000000
O	2.350513	1.089164	0.000000
H	2.585425	-1.330466	0.000000

Compound **35**
E = -539.446720144 a.u. (all vibrational frequencies real)

N	-0.686990	-1.427259	0.002509
C	-1.650554	-0.569536	-0.007540
N	-1.469612	0.776696	-0.000853
C	-0.226876	1.456682	0.002164
C	0.846647	0.499995	0.007369
C	0.534394	-0.834605	-0.000679
N	1.723936	-1.480373	-0.002749
C	2.696242	-0.507393	0.002374
N	2.214372	0.678793	0.008605
H	1.852419	-2.468878	-0.010210
H	3.744106	-0.759126	0.001547
O	-0.216595	2.648456	-0.004416
H	-2.264758	1.380073	-0.069539
N	-2.943729	-1.003574	-0.067982
H	-3.034897	-1.981683	0.122387
H	-3.629067	-0.438875	0.392307

Compound **36**
E = -559.286633329 a.u. (all vibrational frequencies real)

N	0.800221	-1.333133	0.000000
C	-0.483317	-1.840833	0.000000
N	-1.475927	-0.893468	0.000000
C	-1.385382	0.510578	0.000000
C	0.000000	0.945829	0.000000
C	0.992444	0.019120	0.000000
N	2.157853	0.700446	0.000000
C	1.806703	2.038663	0.000000
N	0.541730	2.213920	0.000000
H	3.082251	0.326788	0.000000
H	2.556697	2.811752	0.000000
O	-2.371260	1.172670	0.000000
H	-2.406002	-1.265180	0.000000
O	-0.694318	-3.013585	0.000000
H	1.541846	-2.000542	0.000000

Compound **37**
E = -614.345062521 a.u. (all vibrational frequencies real)

N	-0.901278	-1.445751	0.002796
C	-1.956149	-0.694877	-0.006260
N	-1.916433	0.659574	-0.000238
C	-0.745392	1.449415	0.001690
C	0.395011	0.608102	0.007030
C	0.250595	-0.741767	0.000097
N	1.501471	-1.284809	-0.001477
C	2.472145	-0.286612	0.001912
N	1.769990	0.879422	0.007864
H	1.723663	-2.256772	-0.009675
O	-0.828404	2.644890	-0.004221
H	-2.766703	1.183402	-0.063190
N	-3.188378	-1.269514	-0.065568
H	-3.178386	-2.252565	0.119727
H	-3.946896	-0.777288	0.360704
O	3.654776	-0.451268	-0.001245
H	2.202479	1.776224	0.005693

Compound **38**

E = -447.361474206 a.u. (all vibrational frequencies real)

N	-1.116475	-1.421132	0.000000
C	-2.219706	-0.746719	0.000000
N	-2.368749	0.600199	0.000000
C	-1.279523	1.297994	0.000000
C	-0.000000	0.690139	0.000000
C	0.025320	-0.705094	0.000000
N	1.203716	-1.375386	0.000000
C	2.267025	-0.646631	0.000000
C	2.236851	0.784094	0.000000
N	1.127960	1.439544	0.000000
H	-3.142335	-1.311916	0.000000
H	-1.350914	2.379375	0.000000
H	3.224028	-1.155831	0.000000
H	3.164261	1.343103	0.000000

Compound **39**

E = -577.308789427 a.u. (all vibrational frequencies real)

N	0.849088	-1.411808	0.004095
C	1.856098	-0.622550	-0.006038
N	1.767201	0.739177	0.000763
C	0.568463	1.445027	0.001508
C	-0.601225	0.547547	0.007063
C	-0.395884	-0.835632	-0.000950
N	-1.442944	-1.666792	-0.006061
C	-2.627233	-1.119960	-0.002805
C	-2.827085	0.270295	0.006322
N	-1.813912	1.090868	0.010456
H	-3.478772	-1.790110	-0.006906
H	-3.823882	0.690024	0.009681
O	0.565378	2.633023	-0.008587
N	3.120226	-1.129138	-0.065696
H	3.839266	-0.612622	0.399650
H	3.150923	-2.114881	0.105244
H	2.593033	1.298896	-0.074483

Compound **40**

E = -306.360549797 a.u. (all vibrational frequencies real)

C	0.000000	0.669037	2.036841
C	-0.000000	-0.669037	2.036841
C	0.000000	-0.712237	0.517114
C	0.000000	0.712237	0.517114
C	0.000000	1.440097	-0.614399
C	0.000000	0.680423	-1.839138
C	-0.000000	-0.680423	-1.839138
C	-0.000000	-1.440097	-0.614399
H	0.000000	1.416980	2.815430
H	-0.000000	-1.416980	2.815430
H	0.000000	2.521616	-0.635889
H	0.000000	1.211717	-2.782051
H	-0.000000	-1.211717	-2.782051
H	-0.000000	-2.521616	-0.635889

Compound **41**

E = -322.355715806 a.u. (all vibrational frequencies real)

N	-1.122165	1.817554	-0.000000
C	-1.931981	0.834132	-0.000000
C	-0.866607	-0.250988	-0.000000

C	0.000000	0.861425	-0.000000
C	1.340862	0.767199	0.000000
C	1.828818	-0.585995	0.000000
C	0.997493	-1.668841	0.000000
C	-0.433190	-1.525038	0.000000
H	-3.014332	0.877213	-0.000000
H	2.014627	1.611681	0.000000
H	2.898930	-0.747944	0.000000
H	1.423168	-2.663224	0.000000
H	-1.079608	-2.391963	0.000000

Compound **42**
E = -322.357571998 a.u. (all vibrational frequencies real)

C	1.251994	1.689153	0.000000
C	2.027564	0.596495	0.000000
C	0.829048	-0.334129	-0.000000
C	-0.000000	0.828118	0.000000
N	-1.268961	0.889563	0.000000
C	-1.854573	-0.367835	-0.000000
C	-1.170422	-1.538580	-0.000000
C	0.276966	-1.555745	-0.000000
H	1.449894	2.749767	0.000000
H	3.095777	0.443101	0.000000
H	-2.935517	-0.366729	-0.000000
H	-1.717656	-2.470901	-0.000000
H	0.826760	-2.487044	-0.000000

Compound **43**
E = -338.353316477 a.u. (all vibrational frequencies real)

N	-1.117010	1.765227	-0.000000
C	-1.922908	0.776902	0.000000
C	-0.862658	-0.310029	0.000000
C	0.000000	0.811786	-0.000000
N	1.265518	0.852002	-0.000000
C	1.801779	-0.424838	-0.000000
C	1.082688	-1.579438	0.000000
C	-0.361978	-1.554476	0.000000
H	-3.004316	0.814557	0.000000
H	2.882011	-0.462043	-0.000000
H	1.603854	-2.525993	0.000000
H	-0.942640	-2.466564	0.000000

Compound **44**
E = -397.289184079 a.u. (all vibrational frequencies real)

C	-1.487237	0.938110	0.000000
N	-1.751963	-0.444496	0.000000
C	-0.373850	-0.749613	0.000000
C	-0.000000	0.600456	0.000000
C	1.297712	1.004359	0.000000
C	2.260516	-0.034054	0.000000
C	1.877120	-1.358674	0.000000
C	0.522188	-1.774592	0.000000
O	-2.190748	1.876745	0.000000
H	-2.594714	-0.976317	0.000000
H	1.593921	2.043985	0.000000
H	3.312417	0.214794	0.000000
H	2.646234	-2.120479	0.000000
H	0.253177	-2.820425	0.000000

Compound **45**

E = -397.217719705 a.u. (all vibrational frequencies real)

C	-0.827995	2.034657	0.000000
C	-1.969403	1.345355	0.000000
C	-1.165389	0.030405	0.000000
C	-0.000000	0.741864	0.000000
C	1.283777	0.153976	0.000000
N	1.140402	-1.258526	0.000000
C	-0.001831	-1.963732	0.000000
C	-1.235333	-1.369159	0.000000
H	-0.585790	3.086590	0.000000
H	-3.018374	1.591090	0.000000
O	2.391700	0.628443	0.000000
H	2.013840	-1.747709	0.000000
H	0.114440	-3.038984	0.000000
H	-2.143492	-1.949058	0.000000

Table S1. HF/cc-pVDZ Aromaticity Indices for the Benchmark Compounds in Figure 2[a]

Compound[b]	$NICS_{zz}(0)$	$NICS_{zz}(1)$	$NICS_{zz}(2)$	MCI	SA	HOMA	HOMAc
**1**	-16.621	-32.299	-18.802	0.648	0.0000	1.000	0.998
**2**	-14.744	-31.474	-17.819	0.645	0.0011	0.996	0.993
**3**	-12.647	-31.218	-17.318	0.644	0.0049	0.966	0.969
**4**	-11.597	-29.641	-16.526	0.638	0.0012	0.989	0.987
**5**	-13.970	-31.548	-17.238	0.648	0.0008	0.989	0.987
**6**	-6.860	-26.919	-15.055	0.629	0.0000	0.981	0.980
**7**	8.611	-10.231	-9.248	0.503	0.0023	0.450	0.644
**8**	-10.635	-27.461	-16.102	0.626	0.0019	0.971	0.975
**9**	17.530	-1.786	-5.787	0.406	0.0018	0.501	0.628
**10**	-1.629	-20.290	-12.946	0.593	0.0022	0.969	0.972
**11**	15.134	-5.292	-7.268	0.461	0.0145	-0.049	0.450
**12**	14.106	-5.911	-7.202	0.429	0.0049	-0.188	0.405
**13**	-13.433	-29.273	-17.372	0.637	0.0000	0.995	0.997
**14**	-15.870	-31.377	-18.344	0.640	0.0000	0.998	0.997
**15**	115.983	58.670	10.075	0.288	0.0229	-3.721	-1.741
**16**	120.915	50.679	7.556	0.240	0.0252	-1.876	-1.250
**17**	150.545	52.990	7.952	0.187	0.0453	-1.201	-1.409
**18**	124.408	43.701	5.536	0.332	0.0207	-0.364	-0.641
**19**	48.890	11.552	-2.377	0.343	0.0057	-0.660	-0.160
**20**	93.484	37.853	3.836	0.295	0.0209	-1.055	-0.624
**21**	101.978	51.604	7.713	0.298	0.0179	-2.933	-1.275
**22**	111.354	55.568	9.191	0.252	0.0217	-3.446	-1.580
**23**	-12.392	-32.445	-16.265	0.572	0.0012	0.813	0.863
**24**	-14.456	-34.463	-16.262	0.570	0.0046	0.908	0.939
**25**	-12.421	-32.753	-15.573	0.570	0.0037	0.851	0.844
**26**	-6.452	-27.076	-13.889	0.525	0.0044	0.249	0.666
**27**	-6.914	-27.670	-13.446	0.524	0.0088	0.359	0.637
**28**	9.382	-4.251	-7.233	0.491	0.0045	-0.137	0.349
**29** (right)	-13.509	-30.583	-19.331	0.774	0.0010	0.774	0.877
**29** (left)	-13.509	-30.582	-19.331	0.774	0.0010	0.774	0.877
**30** (right)	-11.273	-29.558	-18.435	0.586	0.0009	0.827	0.887
**30** (left)	-14.288	-30.593	-19.153	0.587	0.0026	0.783	0.881
**31** (right)	-11.478	-29.408	-18.232	0.586	0.0009	0.821	0.884

**31** (left)	-13.221	-30.472	-19.218	0.590	0.0028	0.792	0.886
**32** (right)	-13.151	-30.540	-17.649	0.605	0.0011	0.988	0.988
**32** (left)	-3.283	-26.585	-15.043	0.494	0.0040	0.853	0.822
**33** (right)	-3.565	-22.506	-14.617	0.573	0.0013	0.982	0.986
**33** (left)	-2.933	-26.472	-14.691	0.508	0.0039	0.855	0.823
**34** (right)	10.878	-9.042	-9.511	0.471	0.0051	0.691	0.724
**34** (left)	-5.693	-28.047	-14.446	0.538	0.0036	0.870	0.848
**35** (right)	14.559	-5.978	-8.471	0.457	0.0055	0.726	0.746
**35** (left)	-3.191	-26.294	-13.807	0.531	0.0039	0.853	0.826
**36** (right)	18.640	-0.672	-6.215	0.381	0.0014	0.629	0.685
**36** (left)	-2.897	-26.040	-13.546	0.537	0.0046	0.804	0.783
**37** (right)	12.613	-7.340	-8.358	0.481	0.0048	0.793	0.794
**37** (left)	13.425	-10.702	-8.548	0.414	0.0017	0.773	0.741
**38** (right)	-9.335	-28.072	-17.201	0.583	0.0029	0.840	0.869
**38** (left)	-10.440	-28.076	-16.940	0.579	0.0025	0.901	0.912
**39** (right)	-9.187	-27.633	-16.008	0.618	0.0015	0.955	0.963
**39** (left)	18.073	-3.187	-7.539	0.426	0.0059	0.541	0.663
**40** (right)	96.523	43.165	5.158	0.349	0.0102	-1.503	-0.415
**40** (left)	8.782	-9.125	-8.263	0.601	0.0022	0.512	0.713
**41** (right)	104.867	40.022	3.765	0.309	0.0162	-0.710	-0.303
**41** (left)	3.095	-12.887	-9.722	0.610	0.0020	0.581	0.751
**42** (right)	86.027	37.562	4.032	0.354	0.0096	-1.443	-0.379
**42** (left)	12.305	-7.334	-7.311	0.589	0.0050	0.533	0.661
**43** (right)	93.647	34.528	2.714	0.348	0.0157	-0.647	-0.240
**43** (left)	7.788	-10.151	-8.441	0.595	0.0051	0.591	0.690
**44** (right)	55.014	14.724	-3.103	0.334	0.0035	-0.489	-0.010
**44** (left)	-7.695	-23.316	-14.589	0.622	0.0007	0.852	0.911
**45** (right)	108.723	53.985	8.272	0.322	0.0157	-2.135	-0.800
**45** (left)	8.808	-8.397	-6.676	0.536	0.0020	0.815	0.814

[a]$NICS_{zz}$ values in ppm. [b]For the polycyclic compounds **29**–**45**, aromaticity indices are given for the right-most and left-most rings as shown in Figure 2.

3. M06-2X/cc-pVDZ Cartesian Coordinates, Energies and Aromaticity Indices (Table S2) for the Benchmark Compounds in Figure 2

Cartesian coordinates in Å and electronic energies (*E*) in Hartree atomic units (a.u.).

Compound **1**

E = -232.162455637 a.u. (all vibrational frequencies real)

C	-0.000000	1.394638	-0.000000
C	-1.207792	0.697319	-0.000000
C	-1.207792	-0.697319	-0.000000
C	0.000000	-1.394638	-0.000000
C	1.207792	-0.697319	-0.000000
C	1.207792	0.697319	-0.000000
H	-0.000000	2.485503	-0.000000
H	-2.152509	1.242751	-0.000000
H	-2.152509	-1.242751	-0.000000
H	0.000000	-2.485503	-0.000000
H	2.152509	-1.242751	-0.000000
H	2.152509	1.242751	-0.000000

Compound **2**

E = -248.199244315 a.u. (all vibrational frequencies real)

N	-1.199920	0.760138	0.000000
C	-0.000000	1.347254	0.000000
C	1.207778	0.650488	-0.000000
C	1.169940	-0.741132	-0.000000
C	-0.072118	-1.369923	0.000000
C	-1.218161	-0.575548	0.000000
H	-0.003483	2.440890	0.000000
H	2.153518	1.192335	-0.000000
H	2.091465	-1.325021	-0.000000
H	-0.158168	-2.456512	0.000000
H	-2.208528	-1.039489	0.000000

Compound **3**

E = -264.202211956 a.u. (all vibrational frequencies real)

N	-1.261553	-0.596368	-0.000000
N	-1.191778	0.725829	0.000000
C	-0.000000	1.319702	0.000000
C	1.213987	0.627682	0.000000
C	1.141214	-0.751919	-0.000000
C	-0.138941	-1.312396	-0.000000
H	-0.031871	2.411390	0.000000
H	2.163851	1.162509	0.000000
H	2.029447	-1.383885	-0.000000
H	-0.285669	-2.394647	-0.000000

Compound **4**

E = -264.239642586 a.u. (all vibrational frequencies real)

N	1.196783	0.714991	0.000000
C	0.000000	1.305303	0.000000
N	-1.196781	0.714993	0.000000
C	-1.181901	-0.620104	0.000000
C	-0.000001	-1.353858	0.000000
C	1.181900	-0.620105	0.000000
H	0.000002	2.399026	0.000000
H	-2.156505	-1.116706	0.000000

H	-0.000001	-2.442908	0.000000
H	2.156503	-1.116709	0.000000

Compound **5**
E = -264.232591807 a.u. (all vibrational frequencies real)

N	0.000000	1.409902	-0.000000
C	-1.128096	0.697885	-0.000000
C	-1.128096	-0.697885	-0.000000
N	0.000000	-1.409902	0.000000
C	1.128096	-0.697885	0.000000
C	1.128096	0.697885	0.000000
H	-2.067622	1.256204	-0.000000
H	-2.067622	-1.256204	-0.000000
H	2.067622	-1.256204	0.000000
H	2.067622	1.256204	0.000000

Compound **6**
E = -280.283393912 a.u. (all vibrational frequencies real)

N	1.188591	0.686249	0.000000
C	0.000000	1.290268	0.000000
N	-1.188605	0.686225	0.000000
C	-1.117405	-0.645134	0.000000
N	0.000014	-1.372475	0.000000
C	1.117405	-0.645134	0.000000
H	-0.000009	2.384439	0.000000
H	-2.064980	-1.192227	0.000000
H	2.064989	-1.192212	0.000000

Compound **7**
E = -323.417969762 a.u. (all vibrational frequencies real)

N	-1.135251	0.240456	-0.000000
C	0.000000	1.067231	-0.000000
C	1.248024	0.317480	-0.000000
C	1.263097	-1.043396	0.000000
C	0.052275	-1.804210	0.000000
C	-1.123621	-1.120659	0.000000
H	2.161356	0.910387	-0.000000
H	2.219922	-1.569194	0.000000
H	0.058659	-2.890917	0.000000
H	-2.098487	-1.607737	0.000000
O	-0.127242	2.278568	-0.000000
H	-2.015403	0.747051	-0.000000

Compound **8**
E = -323.421268797 a.u. (all vibrational frequencies real)

N	-1.182842	0.303569	-0.000000
C	0.000000	0.902689	-0.000000
C	1.228190	0.225124	-0.000000
C	1.192998	-1.158799	0.000000
C	-0.042397	-1.815666	0.000000
C	-1.191265	-1.036160	0.000000
H	2.156767	0.793265	-0.000000
H	2.123569	-1.728332	0.000000
H	-0.110398	-2.902317	0.000000
H	-2.180077	-1.502000	0.000000
O	0.010825	2.249131	-0.000000
H	-0.921727	2.518222	-0.000000

Compound **9**
E = -414.705324094 a.u. (all vibrational frequencies real)

N	-1.139147	-1.024430	-0.000000
C	-1.231772	0.362087	-0.000000
N	0.000000	0.986067	-0.000000
C	1.275216	0.395565	0.000000
C	1.240540	-1.067071	0.000000
C	0.053714	-1.703501	0.000000
H	2.185036	-1.602641	0.000000
H	-0.029079	-2.790244	0.000000
O	-2.289629	0.946442	-0.000000
O	2.270077	1.084554	0.000000
H	-2.026757	-1.511561	-0.000000
H	-0.024938	2.002542	-0.000000

Compound **10**
E = -414.690738921 a.u. (all vibrational frequencies real)

N	1.203680	-1.063591	-0.000000
C	1.108618	0.263667	-0.000000
N	-0.000000	1.003555	0.000000
C	-1.136903	0.322027	0.000000
C	-1.194785	-1.079139	0.000000
C	0.032526	-1.715045	-0.000000
H	-2.144133	-1.607532	0.000000
H	0.093610	-2.806413	-0.000000
O	2.251418	0.955850	-0.000000
O	-2.273696	1.023273	0.000000
H	2.959519	0.292715	-0.000000
H	-2.013265	1.959442	0.000000

Compound **11**
E = -343.263584942 a.u. (all vibrational frequencies real)

O	0.329125	-1.153856	-0.000003
C	1.054781	0.039330	-0.000004
C	0.250570	1.256236	-0.000007
C	-1.101190	1.204464	0.000024
C	-1.775796	-0.065291	0.000044
C	-1.012486	-1.176389	0.000025
O	2.250581	-0.036680	-0.000070
H	0.810089	2.189786	-0.000031
H	-1.685493	2.126161	0.000030
H	-2.859631	-0.145767	0.000066
H	-1.397896	-2.195988	0.000031

Compound **12**
E = -343.249547092 a.u. (all vibrational frequencies real)

O	1.756674	0.000000	-0.000023
C	1.056403	-1.157078	0.000052
C	-0.285458	-1.223116	0.000073
C	-1.102924	0.000000	0.000017
C	-0.285458	1.223116	-0.000066
C	1.056403	1.157077	-0.000080
H	1.723886	-2.017641	0.000091
H	-0.785370	-2.190506	0.000134
O	-2.320526	-0.000000	0.000029
H	-0.785369	2.190506	-0.000115
H	1.723886	2.017641	-0.000138

Compound **13**
E = -287.503561528 a.u. (all vibrational frequencies real)

C	-0.938473	0.000001	-0.010720
C	-0.221796	-1.206017	-0.005955

C	1.169528	-1.200730	0.003735
C	1.878608	0.000001	0.008876
C	1.169529	1.200729	0.003734
C	-0.221797	1.206017	-0.005949
H	-0.767300	-2.152159	-0.013882
H	1.705360	-2.151203	0.008949
H	2.968262	0.000001	0.017410
H	1.705360	2.151203	0.008950
H	-0.767298	2.152161	-0.013865
N	-2.333438	-0.000006	-0.083007
H	-2.761953	-0.834232	0.305635
H	-2.761953	0.834271	0.305525

Compound **14**
E = -324.390136574 a.u. (all vibrational frequencies real)

C	0.000000	0.604071	-0.000000
C	1.215573	-0.090722	-0.000000
C	1.209658	-1.482052	0.000000
C	0.000258	-2.177258	0.000000
C	-1.209302	-1.482262	0.000000
C	-1.215521	-0.090980	0.000000
H	2.152632	0.465562	-0.000000
H	2.154190	-2.026224	-0.000000
H	0.000395	-3.267765	0.000000
H	-2.153725	-2.026633	0.000000
H	-2.152595	0.465269	0.000000
C	-0.000514	2.046003	-0.000000
N	-0.000258	3.204142	-0.000000

Compound **15**
E = -154.620014697 a.u. (all vibrational frequencies real)

C	-0.667104	0.786077	-0.000000
C	0.667104	0.786077	-0.000000
C	0.667104	-0.786077	-0.000000
C	-0.667104	-0.786077	-0.000000
H	-1.436994	1.555911	-0.000000
H	1.436990	1.555916	-0.000000
H	1.436994	-1.555911	-0.000000
H	-1.436990	-1.555916	-0.000000

Compound **16**
E = -170.666163420 a.u. (all vibrational frequencies real)

N	-1.054924	0.236768	-0.000000
C	-0.000000	0.959646	-0.000000
C	1.014112	-0.212194	0.000000
C	-0.082196	-0.967743	0.000000
H	0.052525	2.054884	-0.000000
H	2.090830	-0.366323	0.000000
H	-0.350384	-2.024188	0.000000

Compound **17**
E = -186.677011287 a.u. (all vibrational frequencies real)

N	-1.013346	-0.181434	0.000000
N	-0.098357	-1.024699	0.000000
C	0.977088	0.079774	0.000000
C	-0.000000	0.980334	0.000000
H	2.065260	0.022039	0.000000
H	-0.145865	2.060241	0.000000

Compound **18**

E = -186.718765059 a.u. (all vibrational frequencies real)

C	0.747644	0.500102	-0.000000
N	0.747644	-0.777379	-0.000000
C	-0.747644	-0.500102	-0.000000
N	-0.747644	0.777379	-0.000000
H	1.575039	1.220623	-0.000000
H	-1.575039	-1.220623	-0.000000

Compound **19**

E = -245.945310000 a.u. (all vibrational frequencies real)

N	-0.961434	-0.458096	0.000000
C	0.000000	0.593339	0.000000
C	1.109611	-0.468624	-0.000000
C	0.121711	-1.382233	-0.000000
H	2.191483	-0.469542	-0.000000
H	0.038688	-2.467129	-0.000000
O	-0.114915	1.776018	0.000000
H	-1.968745	-0.519690	0.000000

Compound **20**

E = -245.898684013 a.u. (all vibrational frequencies real)

N	-1.090105	-0.262890	-0.000000
C	-0.000000	0.418181	0.000000
C	0.998706	-0.743831	-0.000000
C	-0.133825	-1.451035	-0.000000
H	2.071641	-0.904221	-0.000000
H	-0.432235	-2.498950	-0.000000
O	0.187460	1.721644	0.000000
H	-0.697639	2.130364	0.000000

Compound **21**

E = -209.969997273 a.u. (all vibrational frequencies real)

C	-0.455159	0.064710	0.011571
C	0.433287	1.080233	0.021068
C	1.560608	0.014254	-0.000907
C	0.690648	-1.001566	0.000248
H	0.386895	2.163393	-0.007930
H	2.649292	0.069775	-0.018061
H	0.742796	-2.086677	-0.031447
N	-1.802978	-0.117680	-0.083591
H	-2.158770	-0.966817	0.342471
H	-2.375668	0.698296	0.108224

Compound **22**

E = -246.851762839 a.u. (all vibrational frequencies real)

C	0.000000	0.103906	0.000000
C	1.321815	-0.117397	0.000000
C	1.071351	-1.654153	0.000000
C	-0.247495	-1.452584	-0.000000
H	2.197560	0.529350	0.000000
H	1.717349	-2.528573	0.000000
H	-1.139869	-2.074587	-0.000000
C	-0.826244	1.255416	-0.000000
N	-1.527372	2.180383	-0.000000

Compound **23**

E = -210.099030530 a.u. (all vibrational frequencies real)

C	-0.332729	-1.121387	0.000000
C	0.982647	-0.712123	0.000000
C	0.982647	0.712123	0.000000

C	-0.332729	1.121387	0.000000
N	-1.118235	-0.000000	0.000000
H	-2.127666	0.000000	0.000000
H	-0.771333	-2.113861	0.000000
H	1.849485	-1.365793	0.000000
H	1.849485	1.365793	0.000000
H	-0.771333	2.113861	0.000000

Compound **24**

E = -226.130816260 a.u. (all vibrational frequencies real)

N	0.000000	1.080390	0.000000
N	-1.139039	0.384331	0.000000
C	-0.748131	-0.883983	0.000000
C	0.661928	-1.002112	0.000000
C	1.109649	0.303106	0.000000
H	-0.042025	2.090577	0.000000
H	-1.495631	-1.672844	0.000000
H	1.263389	-1.904249	0.000000
H	2.106861	0.731404	0.000000

Compound **25**

E = -226.148571393 a.u. (all vibrational frequencies real)

N	0.000000	1.102295	0.000000
C	-1.086217	0.280623	0.000000
N	-0.741065	-0.985149	0.000000
C	0.632602	-0.982335	0.000000
C	1.117179	0.301991	0.000000
H	-0.013484	2.112860	0.000000
H	-2.103386	0.663836	0.000000
H	1.201173	-1.907908	0.000000
H	2.121770	0.709513	0.000000

Compound **26**

E = -229.949870463 a.u. (all vibrational frequencies real)

O	-0.000000	1.154234	0.000051
C	1.088580	0.349776	-0.000023
C	0.718052	-0.958674	-0.000013
C	-0.718052	-0.958674	0.000034
C	-1.088580	0.349776	-0.000056
H	2.048922	0.853588	-0.000028
H	1.381801	-1.817137	-0.000016
H	-1.381801	-1.817138	0.000064
H	-2.048923	0.853588	-0.000078

Compound **27**

E = -246.000962583 a.u. (all vibrational frequencies real)

O	-0.356092	-1.079887	0.000023
C	-1.094859	0.045994	-0.000090
N	-0.413135	1.142531	-0.000092
C	0.907884	0.713497	0.000037
C	0.931935	-0.641992	0.000112
H	-2.173884	-0.076468	-0.000172
H	1.738316	1.412082	0.000068
H	1.706490	-1.399234	0.000215

Compound **28**

E = -345.418176293 a.u. (all vibrational frequencies real)

C	-0.000000	-0.000000	1.329374
C	0.000000	1.288657	0.611572
C	0.000000	1.565932	-0.713949

C	0.000000	0.678357	-1.848810
C	-0.000000	-0.678357	-1.848810
C	-0.000000	-1.565932	-0.713949
C	-0.000000	-1.288657	0.611572
O	-0.000000	-0.000000	2.551653
H	0.000000	2.130340	1.308800
H	0.000000	2.628216	-0.972385
H	0.000000	1.169910	-2.824028
H	-0.000000	-1.169910	-2.824028
H	-0.000000	-2.628216	-0.972385
H	-0.000000	-2.130340	1.308800

Compound **29**

E = -385.762449880 a.u. (all vibrational frequencies real)

C	1.243891	-1.400851	0.000000
C	2.429641	-0.709070	0.000000
C	2.429641	0.709070	0.000000
C	1.243891	1.400851	0.000000
C	0.000000	0.712238	0.000000
C	0.000000	-0.712238	0.000000
C	-1.243891	-1.400851	0.000000
C	-2.429640	-0.709070	0.000000
C	-2.429640	0.709070	0.000000
C	-1.243891	1.400851	0.000000
H	1.238411	-2.492736	0.000000
H	3.377573	-1.248387	0.000000
H	3.377573	1.248387	0.000000
H	1.238411	2.492736	0.000000
H	-1.238411	-2.492736	0.000000
H	-3.377573	-1.248387	0.000000
H	-3.377573	1.248387	0.000000
H	-1.238411	2.492736	0.000000

Compound **30**

E = -401.801202560 a.u. (all vibrational frequencies real)

C	1.264801	1.347666	0.000000
C	2.423355	0.611479	0.000000
C	2.375307	-0.806706	-0.000000
C	1.167376	-1.459090	-0.000000
C	-0.047294	-0.722564	-0.000000
C	-0.000000	0.699853	0.000000
N	-1.121602	1.477762	0.000000
C	-2.281664	0.865361	0.000000
C	-2.445310	-0.545704	-0.000000
C	-1.326450	-1.336582	-0.000000
H	1.268277	2.437766	0.000000
H	3.390338	1.115854	0.000000
H	3.304548	-1.377398	-0.000000
H	1.121885	-2.550031	-0.000000
H	-3.167270	1.508366	0.000000
H	-3.447118	-0.974863	-0.000000
H	-1.400181	-2.426307	-0.000000

Compound **31**

E = -401.799344516 a.u. (all vibrational frequencies real)

C	1.240564	1.382560	-0.000000
C	2.419695	0.679263	-0.000000
C	2.404289	-0.739949	0.000000
C	1.216675	-1.429482	0.000000
C	-0.017697	-0.725410	0.000000

C	0.000000	0.693293	-0.000000
C	-1.252400	1.370873	-0.000000
N	-2.420607	0.776302	-0.000000
C	-2.422766	-0.586136	0.000000
C	-1.288119	-1.358004	0.000000
H	1.239520	2.474393	-0.000000
H	3.374024	1.206600	-0.000000
H	3.349169	-1.284934	0.000000
H	1.204445	-2.520804	0.000000
H	-1.256464	2.466979	-0.000000
H	-3.410863	-1.051516	0.000000
H	-1.357013	-2.446883	0.000000

Compound **32**
E = -411.837623625 a.u. (all vibrational frequencies real)

N	-1.265743	1.100028	-0.000000
C	-2.114814	0.071445	-0.000000
N	-1.830845	-1.243068	-0.000000
C	-0.549211	-1.605889	-0.000000
C	0.453088	-0.635670	0.000000
C	0.000000	0.697354	-0.000000
N	1.142933	1.456267	0.000000
C	2.195226	0.568290	0.000000
N	1.836170	-0.686265	0.000000
H	-3.176842	0.327710	-0.000000
H	-0.320646	-2.675018	0.000000
H	1.188633	2.467143	0.000000
H	3.225515	0.918255	0.000000

Compound **33**
E = -467.197867585 a.u. (all vibrational frequencies real)

N	-0.016774	1.922458	-0.001014
C	1.293454	1.690961	0.004364
N	1.949077	0.517949	0.005347
C	1.226332	-0.608022	-0.006121
C	-0.181535	-0.517499	-0.006292
C	-0.705388	0.774601	-0.003805
N	-2.066108	0.590894	0.001198
C	-2.281347	-0.768691	0.004516
N	-1.179904	-1.472388	0.000549
H	1.933366	2.577472	0.011364
H	-2.762815	1.324401	0.004169
H	-3.287289	-1.181759	0.010217
N	1.861440	-1.802748	-0.044627
H	2.854840	-1.806157	0.145520
H	1.318695	-2.635212	0.142583

Compound **34**
E = -487.063989181 a.u. (all vibrational frequencies real)

N	-0.608815	-1.787651	-0.000000
C	0.668904	-2.013095	-0.000000
N	1.631776	-1.049532	-0.000000
C	1.425008	0.361473	-0.000000
C	0.000000	0.625217	0.000000
C	-0.881035	-0.450467	0.000000
N	-2.124793	0.113922	0.000000
C	-1.939166	1.475869	0.000000
N	-0.677445	1.819823	0.000000
H	1.030076	-3.044114	-0.000000
H	-3.000758	-0.393081	0.000000

H	-2.778718	2.166312	0.000000
O	2.368998	1.115636	-0.000000
H	2.610085	-1.324123	-0.000000

Compound **35**

E = -542.417192410 a.u. (all vibrational frequencies real)

N	-0.695298	-1.444502	0.005769
C	-1.664000	-0.568715	-0.005446
N	-1.471337	0.785630	-0.004384
C	-0.216728	1.470756	0.002970
C	0.854793	0.502393	0.008861
C	0.528737	-0.848667	-0.000806
N	1.728696	-1.498194	-0.003945
C	2.707466	-0.525332	0.001778
N	2.219718	0.684703	0.009283
H	1.852397	-2.502307	-0.011802
H	3.763558	-0.781978	0.000307
O	-0.198907	2.679460	-0.004083
H	-2.272694	1.405712	-0.079170
N	-2.970526	-0.999388	-0.074978
H	-3.046238	-1.993636	0.116937
H	-3.646140	-0.443824	0.440036

Compound **36**

E = -562.290393005 a.u. (all vibrational frequencies real)

N	0.808106	-1.343996	0.000000
C	-0.487554	-1.857882	0.000000
N	-1.479683	-0.892829	0.000000
C	-1.390852	0.523438	0.000000
C	0.000000	0.955239	0.000000
C	1.004152	0.011887	0.000000
N	2.173747	0.704682	0.000000
C	1.821452	2.048209	0.000000
N	0.533414	2.224801	0.000000
H	3.112152	0.326683	0.000000
H	2.577023	2.828708	0.000000
O	-2.389825	1.198051	0.000000
H	-2.425241	-1.266174	0.000000
O	-0.703916	-3.047384	0.000000
H	1.553712	-2.028508	0.000000

Compound **37**

E = -617.648654133 a.u. (all vibrational frequencies real)

N	-0.912286	-1.463225	0.006653
C	-1.973247	-0.695396	-0.004611
N	-1.922757	0.668558	-0.004542
C	-0.740496	1.463305	0.002290
C	0.397377	0.607235	0.008537
C	0.246852	-0.761002	0.000178
N	1.507010	-1.302671	-0.002786
C	2.488190	-0.295978	0.000973
N	1.762056	0.879377	0.008322
H	1.736449	-2.287580	-0.010217
O	-0.812066	2.677272	-0.003624
H	-2.780658	1.208573	-0.074816
N	-3.221661	-1.267894	-0.074426
H	-3.190648	-2.265117	0.114050
H	-3.963801	-0.787950	0.424287
O	3.688146	-0.448497	-0.001312
H	2.191435	1.793869	0.009439

Compound **38**

E = -449.910986707 a.u. (all vibrational frequencies real)

N	-1.117706	-1.440003	0.000000
C	-2.232615	-0.751218	0.000000
N	-2.388071	0.605378	0.000000
C	-1.282841	1.307644	0.000000
C	0.000000	0.698340	0.000000
C	0.028818	-0.713998	0.000000
N	1.210210	-1.393412	0.000000
C	2.283028	-0.643471	0.000000
C	2.247734	0.785411	0.000000
N	1.126397	1.460922	0.000000
H	-3.163652	-1.324090	0.000000
H	-1.348667	2.400474	0.000000
H	3.249906	-1.155970	0.000000
H	3.181860	1.353152	0.000000

Compound **39**

E = -580.498446366 a.u. (all vibrational frequencies real)

N	0.851817	-1.429841	0.007245
C	1.868537	-0.624500	-0.003564
N	1.775590	0.745752	-0.004128
C	0.566377	1.457435	0.001619
C	-0.602653	0.550625	0.007754
C	-0.394095	-0.848195	-0.001487
N	-1.447810	-1.690506	-0.007848
C	-2.643012	-1.127288	-0.003902
C	-2.838621	0.266733	0.006829
N	-1.818360	1.108303	0.011728
H	-3.504354	-1.801804	-0.009723
H	-3.844430	0.692007	0.010692
O	0.558884	2.663138	-0.007480
N	3.138639	-1.135913	-0.067101
H	3.870924	-0.614122	0.401800
H	3.158035	-2.135075	0.111543
H	2.610422	1.320461	-0.077238

Compound **40**

E = -308.258776801 a.u. (all vibrational frequencies real)

C	0.000000	0.674553	2.045209
C	-0.000000	-0.674553	2.045209
C	0.000000	-0.711514	0.521845
C	0.000000	0.711514	0.521845
C	0.000000	1.445059	-0.623223
C	0.000000	0.687738	-1.842483
C	-0.000000	-0.687738	-1.842483
C	-0.000000	-1.445059	-0.623223
H	0.000000	1.430754	2.828484
H	-0.000000	-1.430754	2.828484
H	0.000000	2.535147	-0.642550
H	0.000000	1.220989	-2.794025
H	-0.000000	-1.220989	-2.794025
H	-0.000000	-2.535147	-0.642550

Compound **41**

E = -324.297906078 a.u. (all vibrational frequencies real)

N	-1.126940	1.844570	0.000000
C	-1.938345	0.839395	0.000000
C	-0.872602	-0.247216	0.000000

C	-0.000000	0.859939	0.000000
C	1.354507	0.760179	0.000000
C	1.837059	-0.589473	0.000000
C	0.992011	-1.680107	0.000000
C	-0.432799	-1.535794	0.000000
H	-3.031216	0.882877	0.000000
H	2.032199	1.612729	0.000000
H	2.914829	-0.758155	0.000000
H	1.420290	-2.682827	0.000000
H	-1.086501	-2.408158	0.000000

Compound **42**
E = -324.298316695 a.u. (all vibrational frequencies real)

C	1.249814	1.706682	0.000000
C	2.035584	0.608221	0.000000
C	0.834578	-0.326624	0.000000
C	-0.000000	0.828328	0.000000
N	-1.289373	0.888274	-0.000000
C	-1.861262	-0.375002	-0.000000
C	-1.158398	-1.554448	-0.000000
C	0.280453	-1.565069	0.000000
H	1.444982	2.777429	0.000000
H	3.113109	0.454540	0.000000
H	-2.952212	-0.386340	-0.000000
H	-1.706738	-2.496410	-0.000000
H	0.841858	-2.499664	0.000000

Compound **43**
E = -340.337837713 a.u. (all vibrational frequencies real)

N	-1.126748	1.793832	0.000000
C	-1.931184	0.782834	0.000000
C	-0.868583	-0.306894	0.000000
C	-0.000000	0.808323	0.000000
N	1.284189	0.851568	0.000000
C	1.812408	-0.429533	0.000000
C	1.075835	-1.592686	0.000000
C	-0.360523	-1.566468	0.000000
H	-3.023440	0.816001	0.000000
H	2.902406	-0.475317	0.000000
H	1.600208	-2.547762	0.000000
H	-0.948978	-2.484180	0.000000

Compound **44**
E = -399.566074932 a.u. (all vibrational frequencies real)

C	-1.503338	0.931589	0.000000
N	-1.758672	-0.463116	0.000000
C	-0.369179	-0.750647	0.000000
C	0.000000	0.606802	0.000000
C	1.306079	1.017066	0.000000
C	2.272056	-0.020571	0.000000
C	1.894936	-1.356876	0.000000
C	0.540497	-1.777911	0.000000
O	-2.225815	1.876780	0.000000
H	-2.605292	-1.016080	0.000000
H	1.599529	2.066570	0.000000
H	3.331600	0.233983	0.000000
H	2.673824	-2.120974	0.000000
H	0.271267	-2.832641	0.000000

Compound **45**

E = -399.501960979 a.u. (all vibrational frequencies real)

C	-0.823926	2.043610	0.000000
C	-1.975328	1.350562	0.000000
C	-1.175165	0.027781	0.000000
C	0.000000	0.747456	0.000000
C	1.293080	0.161935	0.000000
N	1.133604	-1.269334	0.000000
C	-0.009554	-1.981687	0.000000
C	-1.250115	-1.366406	0.000000
H	-0.571378	3.102174	0.000000
H	-3.031958	1.605744	0.000000
O	2.419126	0.631083	0.000000
H	2.025207	-1.758335	0.000000
H	0.104663	-3.066271	0.000000
H	-2.168725	-1.946148	0.000000

Table S2. M06-2X/cc-pVDZ Aromaticity Indices for the Benchmark Compounds in Figure 2[a]

Compound[b]	$NICS_{zz}(0)$	$NICS_{zz}(1)$	$NICS_{zz}(2)$	MCI	SA	HOMA	HOMAc
**1**	-14.501	-30.265	-17.828	0.652	0.0000	0.989	0.999
**2**	-13.316	-29.771	-16.995	0.650	0.0013	0.995	0.999
**3**	-11.158	-29.281	-16.424	0.650	0.0042	0.985	0.995
**4**	-11.597	-28.824	-16.050	0.647	0.0012	0.999	1.000
**5**	-12.186	-29.324	-16.249	0.651	0.0010	0.995	0.999
**6**	-8.587	-27.159	-14.960	0.643	0.0000	1.000	1.000
**7**	7.087	-11.326	-9.440	0.542	0.0018	0.573	0.698
**8**	-9.823	-26.317	-15.487	0.633	0.0018	0.983	0.992
**9**	15.935	-2.580	-5.818	0.453	0.0016	0.500	0.590
**10**	-3.536	-20.937	-12.954	0.608	0.0019	0.988	0.993
**11**	12.987	-6.680	-7.579	0.509	0.0041	-0.051	0.333
**12**	13.086	-6.515	-7.227	0.473	0.0042	-0.166	0.402
**13**	-10.696	-26.847	-16.215	0.638	0.0000	0.976	0.994
**14**	-13.764	-29.312	-17.337	0.643	0.0000	0.982	0.996
**15**	127.588	66.717	12.056	0.327	0.0233	-3.742	-1.745
**16**	130.087	56.622	9.131	0.256	0.0271	-2.035	-1.472
**17**	165.947	60.523	10.040	0.199	0.0457	-1.377	-1.720
**18**	131.268	48.230	6.913	0.288	0.0263	-0.781	-1.144
**19**	55.800	17.271	-0.963	0.368	0.0065	-0.906	-0.342
**20**	98.894	42.438	5.010	0.300	0.0243	-1.429	-0.973
**21**	113.069	59.735	9.613	0.311	0.0184	-3.010	-1.312
**22**	121.882	62.994	11.031	0.321	0.0218	-3.453	-1.574
**23**	-12.748	-32.710	-16.254	0.598	0.0006	0.875	0.897
**24**	-15.499	-34.589	-16.197	0.593	0.0044	0.933	0.962
**25**	-14.457	-33.569	-15.737	0.596	0.0027	0.904	0.885
**26**	-9.123	-28.798	-14.354	0.564	0.0028	0.305	0.678
**27**	-11.323	-29.784	-14.000	0.563	0.0067	0.359	0.645
**28**	8.831	-4.673	-7.176	0.529	0.0035	0.117	0.504
**29** (right)	-12.878	-29.853	-18.835	0.589	0.0010	0.774	0.877
**29** (left)	-12.878	-29.852	-18.835	0.589	0.0010	0.774	0.877
**30** (right)	-11.255	-29.069	-18.071	0.596	0.0007	0.824	0.896
**30** (left)	-13.932	-30.115	-18.767	0.593	0.0025	0.793	0.895
**31** (right)	-11.775	-29.160	-17.941	0.593	0.0007	0.830	0.901

**31** (left)	-12.778	-29.916	-18.815	0.597	0.0027	0.809	0.904
**32** (right)	-13.931	-30.362	-17.570	0.978	0.0014	0.978	0.990
**32** (left)	-8.056	-29.657	-15.888	0.856	0.0031	0.856	0.839
**33** (right)	-4.375	-22.453	-14.507	0.974	0.0015	0.974	0.988
**33** (left)	-6.710	-28.923	-15.352	0.880	0.0028	0.880	0.854
**34** (right)	8.646	-10.366	-9.837	0.659	0.0048	0.659	0.697
**34** (left)	-8.359	-29.508	-14.827	0.908	0.0026	0.908	0.887
**35** (right)	12.234	-7.378	-8.789	0.680	0.0051	0.680	0.705
**35** (left)	-5.714	-27.793	-14.189	0.892	0.0029	0.892	0.867
**36** (right)	17.030	-1.590	-6.325	0.567	0.0012	0.567	0.608
**36** (left)	-4.664	-27.011	-13.751	0.870	0.0035	0.870	0.841
**37** (right)	10.397	-8.656	-8.634	0.780	0.0043	0.780	0.773
**37** (left)	10.376	-12.707	-9.001	0.766	0.0007	0.766	0.114
**38** (right)	-10.332	-28.161	-17.105	0.593	0.0028	0.857	0.902
**38** (left)	-11.746	-28.374	-16.907	0.589	0.0026	0.888	0.919
**39** (right)	-7.964	-25.873	-15.224	0.620	0.0019	0.945	0.971
**39** (left)	16.063	-4.376	-7.645	0.460	0.0057	0.464	0.612
**40** (right)	109.289	52.434	7.450	0.392	0.0105	-1.553	-0.440
**40** (left)	10.988	-7.114	-7.095	0.622	0.0014	0.680	0.820
**41** (right)	116.026	48.015	5.762	0.328	0.0170	-0.846	-0.455
**41** (left)	4.441	-11.440	-8.793	0.626	0.0013	0.737	0.850
**42** (right)	98.766	47.105	6.382	0.383	0.0103	-1.590	-0.460
**42** (left)	13.367	-6.102	-6.414	0.614	0.0039	0.706	0.788
**43** (right)	104.361	42.541	4.673	0.350	0.0170	-0.862	-0.465
**43** (left)	7.565	-9.803	-7.899	0.618	0.0041	0.748	0.807
**44** (right)	60.589	19.224	-1.827	0.338	0.0043	-0.806	-0.234
**44** (left)	-4.833	-20.676	-13.275	0.629	0.0005	0.881	0.936
**45** (right)	119.116	61.194	10.063	0.362	0.0154	-2.148	-0.799
**45** (left)	10.341	-6.686	-5.599	0.555	0.0021	0.772	0.756

[a]$NICS_{zz}$ values in ppm. [b]For the polycyclic compounds **29**–**45**, aromaticity indices are given for the right-most and left-most rings as shown in Figure 2.

4. CASPT2/cc-pVDZ Cartesian Coordinates, Energies and Aromaticity Indices (Table S3) for the Benchmark Compounds in Figure 2

Cartesian coordinates in Å and electronic energies (*E*) in Hartree atomic units (a.u.).

Compound **1**
E = -231.50062660 a.u. (all vibrational frequencies real)

C	-1.21803500	0.70323300	0.00000000
C	0.00000000	1.40646500	0.00000000
C	1.21803500	0.70323300	0.00000000
C	1.21803500	-0.70323200	0.00000000
C	0.00000000	-1.40646500	0.00000000
C	-1.21803400	-0.70323300	0.00000000
H	-2.16497700	1.24995000	0.00000000
H	0.00000000	2.49990100	0.00000000
H	2.16497700	1.24995000	0.00000000
H	2.16497800	-1.24995000	0.00000000
H	0.00000000	-2.49990100	0.00000000
H	-2.16497700	-1.24995100	0.00000000

Compound **2**
E = -247.51718473 a.u. (all vibrational frequencies real)

N	-1.21502200	-0.76925700	0.00000000
C	-0.00079700	-1.35646400	0.00000000
C	1.21772600	-0.65478200	0.00000000
C	1.18220300	0.74847900	0.00000000
C	-0.07103100	1.38077900	0.00000000
C	-1.22646900	0.57945400	0.00000000
H	-0.00237100	-2.45199100	0.00000000
H	2.16589200	-1.19858100	0.00000000
H	2.10629000	1.33353700	0.00000000
H	-0.15704300	2.47043000	0.00000000
H	-2.21740500	1.04660200	0.00000000

Compound **3**
E = -263.50054248 a.u. (all vibrational frequencies real)

N	1.20632100	-0.73552100	0.00000000
N	1.27675900	0.60505100	0.00000000
C	0.13677400	1.32181500	0.00000000
C	-1.15288000	0.75888900	0.00000000
C	-1.22606100	-0.63389200	0.00000000
C	-0.00250200	-1.32887100	0.00000000
H	0.28114400	2.40636400	0.00000000
H	-2.04374700	1.39190600	0.00000000
H	-2.17836300	-1.17006000	0.00000000
H	0.02741200	-2.42257700	0.00000000

Compound **4**
E = -263.53762102 a.u. (all vibrational frequencies real)

N	-1.21118600	-0.72391500	0.00000000
C	0.00149600	-1.31087200	0.00000000
N	1.21283000	-0.72115500	0.00000000
C	1.19159700	0.62639300	0.00000000
C	-0.00155200	1.36336300	0.00000000
C	-1.19302200	0.62367300	0.00000000
H	0.00274200	-2.40569500	0.00000000
H	2.16570300	1.12763200	0.00000000

H	-0.00280000	2.45551800	0.00000000
H	-2.16826700	1.12269300	0.00000000

Compound **5**

E = -263.53005695 a.u. (all vibrational frequencies real)

N	-0.00000300	1.43048200	0.00000000
C	1.13442400	0.70304400	0.00000000
C	1.13442700	-0.70303900	0.00000000
N	0.00000300	-1.43048100	0.00000000
C	-1.13442400	-0.70304400	0.00000000
C	-1.13442700	0.70303900	0.00000000
H	2.07753400	1.25918900	0.00000000
H	2.07753900	-1.25918000	0.00000000
H	-2.07753500	-1.25918800	0.00000000
H	-2.07754000	1.25917900	0.00000000

Compound **6**

E = -279.56176257 a.u. (all vibrational frequencies real)

N	-1.20247400	-0.69425600	0.00000000
C	-1.12413200	0.64901300	0.00000000
N	-0.00000600	1.38850100	0.00000000
C	1.12412700	0.64902000	0.00000000
N	1.20248000	-0.69424500	0.00000000
C	0.00000500	-1.29803300	0.00000000
H	-2.07244800	1.19651500	0.00000000
H	2.07243600	1.19653500	0.00000000
H	0.00001200	-2.39305000	0.00000000

Compound **7**

E = -322.57704740 a.u. (all vibrational frequencies real)

N	-1.13718600	-0.24181700	0.00000000
C	-0.00030200	-1.07606100	0.00000000
C	1.24869600	-0.31942400	0.00000000
C	1.27038500	1.05860600	0.00000000
C	0.05579200	1.81747700	0.00000000
C	-1.13361800	1.12654400	0.00000000
H	2.16801400	-0.90925100	0.00000000
H	2.23102800	1.58281600	0.00000000
H	0.05752900	2.90757800	0.00000000
H	-2.11181100	1.61214500	0.00000000
O	-0.12615200	-2.29963700	0.00000000
H	-2.02095900	-0.74632800	0.00000000

Compound **8**

E = -322.57900961 a.u. (all vibrational frequencies real)

N	-1.19752400	-0.30415000	0.00000000
C	-0.00507900	-0.90895500	0.00000000
C	1.23471200	-0.23465100	0.00000000
C	1.21011500	1.16217000	0.00000000
C	-0.03166700	1.82839100	0.00000000
C	-1.19415100	1.04854900	0.00000000
H	2.16372100	-0.80756300	0.00000000
H	2.14659600	1.72716700	0.00000000
H	-0.09491800	2.91849800	0.00000000
H	-2.18141900	1.52220600	0.00000000
O	0.00071900	-2.26245200	0.00000000
H	-0.94064100	-2.50468500	0.00000000

Compound **9**

E = -413.68292761 a.u. (all vibrational frequencies real)

N	-1.14453000	1.02974800	0.00000000
C	-1.24083900	-0.35733500	0.00000000
N	-0.00160000	-0.98172700	0.00000000
C	1.28363100	-0.40112100	0.00000000
C	1.24949400	1.06547200	0.00000000
C	0.05259800	1.71373900	0.00000000
H	2.19433700	1.60700300	0.00000000
H	-0.02837500	2.80279900	0.00000000
O	-2.30560100	-0.94960000	0.00000000
O	2.28675400	-1.09945300	0.00000000
H	-2.03052000	1.52186900	0.00000000
H	-0.03105600	-1.99992600	0.00000000

Compound **10**
E = -413.66840757 a.u. (all vibrational frequencies real)

N	1.21553600	1.07594500	0.00000000
C	1.11399600	-0.26194800	0.00000000
N	0.00204900	-1.01456900	0.00000000
C	-1.14412100	-0.32668500	0.00000000
C	-1.20429300	1.08124300	0.00000000
C	0.03066600	1.72693700	0.00000000
H	-2.15580100	1.61204700	0.00000000
H	0.08664500	2.82045400	0.00000000
O	2.26178700	-0.95846100	0.00000000
O	-2.28524600	-1.03373900	0.00000000
H	2.95525800	-0.27800400	0.00000000
H	-1.99901400	-1.96380500	0.00000000

Compound **11**
E = -342.39640884 a.u. (all vibrational frequencies real)

O	0.33241900	-1.16944500	-0.00000500
C	1.05843200	0.02823200	-0.00002600
C	0.25860400	1.25616200	-0.00000900
C	-1.10929500	1.22148300	0.00002300
C	-1.78825200	-0.05100700	0.00004300
C	-1.02407700	-1.17835300	0.00002800
O	2.26504400	-0.03959400	-0.00005400
H	0.82523000	2.18898300	-0.00002400
H	-1.68424600	2.15180000	0.00003500
H	-2.87526500	-0.13128600	0.00006900
H	-1.41789600	-2.19628200	0.00003900

Compound **12**
E = -342.38168413 a.u. (all vibrational frequencies real)

O	1.78224600	-0.00000800	0.00002900
C	1.06547100	1.15885800	-0.00005400
C	-0.29197200	1.22303600	-0.00007900
C	-1.11221000	0.00000400	-0.00001400
C	-0.29198400	-1.22303500	0.00005600
C	1.06546100	-1.15886500	0.00007700
H	1.72660300	2.02667100	-0.00009100
H	-0.78492800	2.19714200	-0.00015400
O	-2.34373500	0.00001200	-0.00001700
H	-0.78493800	-2.19714200	0.00010300
H	1.72658200	-2.02668700	0.00013400

Compound **13**
E = -286.70468396 a.u. (all vibrational frequencies real)

C	-1.23528700	0.71319300	0.00040600
C	-0.00451800	1.40523200	0.00002800

C	1.20936300	0.70038000	-0.02980400
C	1.22161200	-0.70534000	-0.05303300
C	-0.00219800	-1.39741700	-0.04770400
C	-1.21956500	-0.69859200	-0.01802800
H	-0.00334600	2.50006300	0.02327200
H	2.15131400	1.25685600	-0.03347700
H	2.16793800	-1.25168200	-0.07619500
H	-0.01340000	-2.49127600	-0.06545700
H	-2.16739800	-1.24694400	-0.00893000
N	-2.45211900	1.41511600	0.10671700
H	-2.39606800	2.34076300	-0.31431700
H	-3.22284700	0.90915600	-0.32591900

Compound **14**
E = -323.50333548 a.u. (all vibrational frequencies real)

C	-1.23217700	0.71139800	0.00000000
C	-0.01903300	1.42289500	0.00000000
C	1.19690200	0.72290400	0.00000000
C	1.20120000	-0.68853500	0.00000000
C	-0.01500400	-1.39527900	0.00000000
C	-1.23545500	-0.70182800	0.00000000
H	-0.03418200	2.51626300	0.00000000
H	2.14038200	1.27602100	0.00000000
H	2.15073200	-1.23137400	0.00000000
H	-0.01447700	-2.48858000	0.00000000
H	-2.18690900	-1.23912900	0.00000000
C	-2.48497900	1.43003900	0.00000000
N	-3.50909900	2.01704700	0.00000000

Compound **15**
E = -154.18587430 a.u. (all vibrational frequencies real)

C	0.67081900	0.79191100	0.00000000
C	-0.69925200	0.76691700	0.00000000
C	-0.67081900	-0.79191100	0.00000000
C	0.69925200	-0.76691700	0.00000000
H	1.42866500	1.57654500	0.00000000
H	-1.48519300	1.52340800	0.00000000
H	-1.42866500	-1.57654500	0.00000000
H	1.48519300	-1.52340800	0.00000000

Compound **16**
E = -170.21132921 a.u. (all vibrational frequencies real)

N	-1.06163100	-0.23859000	0.00000000
C	0.02409100	-0.97050800	0.00000000
C	1.01930900	0.21165000	0.00000000
C	-0.10547200	0.97946700	0.00000000
H	0.08923300	-2.06408200	0.00000000
H	2.09796600	0.37602900	0.00000000
H	-0.38334800	2.03453500	0.00000000

Compound **17**
E = -186.20540000 a.u. (all vibrational frequencies real)

N	-1.02286500	-0.18338400	0.00000000
N	-0.05810700	-1.03757100	0.00000000
C	0.98368800	0.08543000	0.00000000
C	-0.03437600	0.98679000	0.00000000
H	2.07467500	0.04813000	0.00000000
H	-0.20374700	2.06523300	0.00000000

Compound **18**

E = -186.24240377 a.u. (all vibrational frequencies real)

C	0.59072700	0.69736200	0.00000000
N	-0.70428600	0.82779200	0.00000000
C	-0.59072700	-0.69736200	0.00000000
N	0.70428600	-0.82779200	0.00000000
H	1.39288600	1.44472600	0.00000000
H	-1.39288600	-1.44472600	0.00000000

Compound **19**

E = -245.32529824 a.u. (all vibrational frequencies real)

N	0.01192500	0.00000000	0.03188900
C	-0.03080600	0.00000000	1.45846900
C	1.51540100	0.00000000	1.46895600
C	1.44264000	0.00000000	0.10543700
H	2.29690500	0.00000000	2.22177000
H	2.13457600	0.00000000	-0.73656900
O	-0.93978400	0.00000000	2.24228400
H	-0.67406100	0.00000000	-0.71015000

Compound **20**

E = -245.28035878 a.u. (all vibrational frequencies real)

N	1.10045500	-0.27385900	0.00000000
C	0.00265100	0.42489800	0.00000000
C	-1.00743000	-0.73504600	0.00000000
C	0.13048500	-1.47533900	0.00000000
H	-2.08554600	-0.88589500	0.00000000
H	0.41984800	-2.52678300	0.00000000
O	-0.18727500	1.73911700	0.00000000
H	0.70647000	2.12967400	0.00000000

Compound **21**

E = -209.39530977 a.u. (all vibrational frequencies real)

C	0.67878300	0.80235300	0.00076000
C	-0.69126600	0.85703900	0.00663700
C	-0.71734100	-0.69194700	-0.17483700
C	0.64758700	-0.74843300	-0.19193700
H	-1.44012100	1.62885000	0.18128400
H	-1.51302300	-1.43324000	-0.26596400
H	1.39824000	-1.53491800	-0.27884400
N	1.75224400	1.64215800	0.25040200
H	2.54541600	1.44887200	-0.36022400
H	1.50022300	2.62748200	0.18439400

Compound **22**

E = -246.19645112 a.u. (all vibrational frequencies real)

C	0.67072800	0.82025000	0.00000000
C	-0.64568300	0.40353800	0.00000000
C	-0.17882800	-1.06823500	0.00000000
C	1.13404200	-0.67216500	0.00000000
H	-1.60568600	0.92089400	0.00000000
H	-0.69295600	-2.02937700	0.00000000
H	2.10728200	-1.16378700	0.00000000
C	1.32276900	2.07684100	0.00000000
N	1.88780600	3.12424100	0.00000000

Compound **23**

E = -209.52095603 a.u. (all vibrational frequencies real)

C	1.13020400	0.33448000	0.00000000
C	0.71235300	-0.99388500	0.00000000
C	-0.71770700	-0.99002600	0.00000000

C	-1.12838300	0.34057500	0.00000000
N	0.00303800	1.12571300	0.00000000
H	0.00576800	2.13755000	0.00000000
H	2.12578300	0.77299200	0.00000000
H	1.36606100	-1.86462600	0.00000000
H	-1.37611000	-1.85722400	0.00000000
H	-2.12157900	0.78445900	0.00000000

Compound **24**
E = -225.53493742 a.u. (all vibrational frequencies real)

N	0.00000000	1.08682100	0.00000000
N	-1.15325000	0.39020200	0.00000000
C	-0.74748500	-0.89443400	0.00000000
C	0.66561800	-1.01085400	0.00000000
C	1.11886300	0.30901200	0.00000000
H	-0.04267600	2.09919400	0.00000000
H	-1.49408400	-1.68730200	0.00000000
H	1.27018700	-1.91465500	0.00000000
H	2.11735200	0.74125600	0.00000000

Compound **25**
E = -225.55093811 a.u. (all vibrational frequencies real)

N	0.00000000	1.11007600	0.00000000
C	-1.09103900	0.28438000	0.00000000
N	-0.74710700	-0.99861200	0.00000000
C	0.63418900	-0.98895300	0.00000000
C	1.12573300	0.30814100	0.00000000
H	-0.01442300	2.12298900	0.00000000
H	-2.10890900	0.67094700	0.00000000
H	1.20775400	-1.91431500	0.00000000
H	2.13202400	0.71872200	0.00000000

Compound **26**
E = -229.34847278 a.u. (all vibrational frequencies real)

O	0.00001000	-1.16932600	0.00056500
C	-1.09437300	-0.35314500	-0.00027000
C	-0.71953100	0.97042100	-0.00082100
C	0.71951300	0.97043400	-0.00020800
C	1.09437900	-0.35312500	0.00068900
H	-2.05749400	-0.85584600	-0.00034100
H	-1.38740300	1.82936100	-0.00160500
H	1.38737000	1.82938600	-0.00046400
H	2.05750900	-0.85581000	0.00155000

Compound **27**
E = -245.38032570 a.u. (all vibrational frequencies real)

O	0.38724900	1.08554900	0.00002900
C	1.09663200	-0.07166000	-0.00009400
N	0.38534500	-1.16806000	-0.00009400
C	-0.92818300	-0.69995700	0.00003900
C	-0.92090300	0.67094100	0.00010800
H	2.17954800	0.02444000	-0.00017500
H	-1.78055700	-1.37524200	0.00006700
H	-1.67967300	1.44689500	0.00021300

Compound **28**
E = -344.49450737 a.u. (all vibrational frequencies real)

C	0.00000000	1.70720700	0.00000000
C	1.29232400	0.99151800	0.00000000
C	1.57679200	-0.35161800	0.00000000

C	0.68821600	-1.48939300	0.00000000
C	-0.68821600	-1.48939300	0.00000000
C	-1.57679200	-0.35161800	0.00000000
C	-1.29232400	0.99151800	0.00000000
O	0.00000000	2.94499500	0.00000000
H	2.13910400	1.68648200	0.00000000
H	2.64299400	-0.60467700	0.00000000
H	1.17767800	-2.46865700	0.00000000
H	-1.17767800	-2.46865700	0.00000000
H	-2.64299400	-0.60467700	0.00000000
H	-2.13910400	1.68648200	0.00000000

Compound **29**

E = -384.66994290 a.u. (all vibrational frequencies real)

C	1.41241300	0.01661200	0.00000000
C	0.71365000	1.21710000	0.00000000
C	-0.71083900	1.21791800	0.00000000
C	-1.41097900	0.01823200	0.00000000
C	-0.71845600	-1.23158400	0.00000000
C	0.71845500	-1.23240800	0.00000000
C	1.41097900	-2.48222300	0.00000000
C	0.71083900	-3.68190900	0.00000000
C	-0.71365100	-3.68109200	0.00000000
C	-1.41241300	-2.48060300	0.00000000
H	2.50726000	0.01211000	0.00000000
H	1.25664500	2.16659800	0.00000000
H	-1.25274400	2.16803900	0.00000000
H	-2.50583100	0.01498600	0.00000000
H	2.50583000	-2.47897700	0.00000000
H	1.25274300	-4.63203100	0.00000000
H	-1.25664500	-4.63059000	0.00000000
H	-2.50726000	-2.47610200	0.00000000

Compound **30**

E = -400.68827205 a.u. (all vibrational frequencies real)

C	0.06532700	1.41320900	0.00000000
C	1.23814700	0.66962000	0.00000000
C	1.19000300	-0.75499400	0.00000000
C	-0.03226200	-1.41442300	0.00000000
C	-1.25265100	-0.67301500	0.00000000
C	-1.20516100	0.76170200	0.00000000
N	-2.32797000	1.55344200	0.00000000
C	-3.50185800	0.92742100	0.00000000
C	-3.66704100	-0.48787500	0.00000000
C	-2.53670100	-1.29109700	0.00000000
H	0.07139000	2.50609600	0.00000000
H	2.20693900	1.17722500	0.00000000
H	2.12107200	-1.32876900	0.00000000
H	-0.07634200	-2.50833500	0.00000000
H	-4.38847600	1.57239300	0.00000000
H	-4.67183000	-0.91828500	0.00000000
H	-2.61396300	-2.38328200	0.00000000

Compound **31**

E = -400.68645988 a.u. (all vibrational frequencies real)

C	0.02669100	1.41738400	0.00000000
C	1.22016300	0.70691900	0.00000000
C	1.20667300	-0.71889800	0.00000000
C	0.00533700	-1.41684800	0.00000000
C	-1.23474900	-0.70863100	0.00000000

C	-1.21958200	0.72272700	0.00000000
C	-2.47692600	1.40270300	0.00000000
N	-3.66496900	0.80689000	0.00000000
C	-3.65923300	-0.56302600	0.00000000
C	-2.51082600	-1.34354900	0.00000000
H	0.02737200	2.51204300	0.00000000
H	2.17583900	1.23838000	0.00000000
H	2.15399800	-1.26578900	0.00000000
H	-0.00469500	-2.51114900	0.00000000
H	-2.48159000	2.50045400	0.00000000
H	-4.64720500	-1.03442000	0.00000000
H	-2.58257000	-2.43530200	0.00000000

Compound **32**
E = -410.76808301 a.u. (all vibrational frequencies real)

N	-0.38684500	1.38641100	0.00000000
C	-1.23572900	0.33848000	0.00000000
N	-0.95453400	-0.98866800	0.00000000
C	0.34275500	-1.34734200	0.00000000
C	1.34848700	-0.36749400	0.00000000
C	0.88806400	0.97370000	0.00000000
N	2.03319700	1.73619300	0.00000000
C	3.09281400	0.84950400	0.00000000
N	2.73646000	-0.42504500	0.00000000
H	-2.30001000	0.59260600	0.00000000
H	0.57420900	-2.41777400	0.00000000
H	2.07821800	2.74971100	0.00000000
H	4.12293700	1.20562300	0.00000000

Compound **33**
E = -465.99037101 a.u. (all vibrational frequencies real)

N	-0.62920200	1.30181400	-0.00158500
C	0.69659600	1.06860400	0.00884800
N	1.36868300	-0.10769400	0.01181600
C	0.64325800	-1.24469300	-0.00491300
C	-0.76944600	-1.16365700	-0.00702600
C	-1.30612500	0.13708900	-0.00545200
N	-2.67023700	-0.05809400	-0.00022300
C	-2.87837600	-1.42287300	0.00655900
N	-1.76056100	-2.13494400	0.00325700
H	1.33126200	1.96071800	0.01957900
H	-3.37555500	0.67086600	0.00253200
H	-3.88458600	-1.84076200	0.01396100
N	1.29759500	-2.44229400	-0.06988800
H	2.26848400	-2.41324800	0.22397600
H	0.76361500	-3.25246500	0.22602600

Compound **34**
E = -485.83261162 a.u. (all vibrational frequencies real)

N	-0.98854800	-1.08421100	0.00000000
C	0.30764600	-1.29472200	0.00000000
N	1.26324300	-0.31692800	0.00000000
C	1.05361400	1.09522600	0.00000000
C	-0.37518800	1.34942900	0.00000000
C	-1.25978200	0.25818800	0.00000000
N	-2.51133700	0.82146000	0.00000000
C	-2.33247500	2.18933300	0.00000000
N	-1.05720300	2.55062100	0.00000000
H	0.68144700	-2.32309300	0.00000000
H	-3.38846100	0.31129200	0.00000000

H	-3.17930400	2.87411100	0.00000000
O	2.00246100	1.86217700	0.00000000
H	2.24429300	-0.59061100	0.00000000

Compound **35**

E = -541.04846483 a.u. (all vibrational frequencies real)

N	-0.25907500	-1.44120500	0.00664800
C	-1.22458000	-0.54558500	-0.00570500
N	-1.02104700	0.80982200	-0.00685200
C	0.23431800	1.49817500	0.00093900
C	1.30337100	0.52319400	0.00724600
C	0.96949500	-0.83976500	-0.00315900
N	2.17433600	-1.49438000	-0.00749600
C	3.15946700	-0.52299900	-0.00219600
N	2.67550700	0.70817700	0.00661600
H	2.29814600	-2.50106200	-0.01619600
H	4.21633500	-0.78563100	-0.00463200
O	0.25699900	2.71818200	-0.00783400
H	-1.82543600	1.42834700	-0.09349400
N	-2.54788300	-0.96294200	-0.08561500
H	-2.60570600	-1.96121700	0.10711200
H	-3.17605200	-0.44341600	0.52524100

Compound **36**

E = -560.89748360 a.u. (all vibrational frequencies real)

N	1.06595000	-0.91779100	0.00000000
C	-0.23298200	-1.42975500	0.00000000
N	-1.22141400	-0.45376500	0.00000000
C	-1.13633100	0.96647300	0.00000000
C	0.25649700	1.39636800	0.00000000
C	1.26650400	0.44180400	0.00000000
N	2.44334000	1.13533700	0.00000000
C	2.09553500	2.48392800	0.00000000
N	0.79150100	2.67427700	0.00000000
H	3.38322500	0.75447900	0.00000000
H	2.85731900	3.26113600	0.00000000
O	-2.14562500	1.64669900	0.00000000
H	-2.16914300	-0.82651700	0.00000000
O	-0.45850600	-2.62748600	0.00000000
H	1.81282700	-1.60309800	0.00000000

Compound **37**

E = -616.12007893 a.u. (all vibrational frequencies real)

N	-0.09876200	-1.44448700	0.00770300
C	-1.16050000	-0.65504900	-0.00480100
N	-1.09829800	0.70963400	-0.00910600
C	0.08654000	1.50933800	0.00092300
C	1.21861600	0.64352300	0.00780800
C	1.06418400	-0.74016700	-0.00220000
N	2.32825900	-1.27994300	-0.00571400
C	3.31606900	-0.27467700	-0.00249800
N	2.58618500	0.90588100	0.00699500
H	2.55727000	-2.26714300	-0.01498400
O	0.01657100	2.73561900	-0.00585900
H	-1.95950900	1.24791100	-0.09244700
N	-2.42849400	-1.21749200	-0.08754900
H	-2.37188300	-2.21816100	0.09324200
H	-3.11096100	-0.78115300	0.53016100
O	4.52541800	-0.42966900	-0.00633100
H	3.01936700	1.82092900	0.00721600

Compound **38**

E = -448.73653007 a.u. (all vibrational frequencies real)

N	0.05211000	-1.40691100	0.00000000
C	-1.07422700	-0.70214500	0.00000000
N	-1.23798900	0.65974000	0.00000000
C	-0.11396000	1.36572600	0.00000000
C	1.17210000	0.75366200	0.00000000
C	1.20186200	-0.67107400	0.00000000
N	2.38571600	-1.36273500	0.00000000
C	3.46862200	-0.59472900	0.00000000
C	3.43292300	0.83696800	0.00000000
N	2.30057000	1.53002000	0.00000000
H	-2.00468700	-1.27875100	0.00000000
H	-0.18112100	2.45974600	0.00000000
H	4.43759000	-1.10711700	0.00000000
H	4.36928300	1.40496000	0.00000000

Compound **39**

E = -579.02366875 a.u. (all vibrational frequencies real)

N	0.17520000	-1.41936400	0.00793700
C	1.19236800	-0.59881200	-0.00384400
N	1.09634700	0.77395600	-0.00513800
C	-0.11096000	1.49148600	0.00022700
C	-1.28085700	0.58129600	0.00721500
C	-1.07461100	-0.82727700	-0.00479500
N	-2.13008800	-1.68448000	-0.01421700
C	-3.33654600	-1.10841500	-0.01080200
C	-3.53085100	0.29323300	0.00313900
N	-2.50623800	1.15247900	0.01095200
H	-4.20080300	-1.78229300	-0.01913900
H	-4.53973000	0.71762000	0.00733300
O	-0.12063500	2.70915100	-0.01262700
N	2.47877100	-1.10212100	-0.08022800
H	3.15798300	-0.61644300	0.50184100
H	2.47449400	-2.10349100	0.10400200
H	1.93614300	1.34275200	-0.09483000

Compound **40**

E = -307.38801163 a.u. (all vibrational frequencies real)

C	0.68480437	2.05617133	0.00000000
C	-0.69335840	2.05330769	0.00000000
C	-0.71866766	0.52819604	0.00000000
C	0.71645433	0.53118034	0.00000000
C	1.46139813	-0.62655323	0.00000000
C	0.70137182	-1.85020243	0.00000000
C	-0.69365650	-1.85310888	0.00000000
C	-1.45877782	-0.63263666	0.00000000
H	1.44305605	2.84142525	0.00000000
H	-1.45487333	2.83540882	0.00000000
H	2.55452253	-0.64400245	0.00000000
H	1.23375482	-2.80559348	0.00000000
H	-1.22204753	-2.81070937	0.00000000
H	-2.55182210	-0.65464800	0.00000000

Compound **41**

E = -323.40743588 a.u. (all vibrational frequencies real)

N	-1.13628324	-1.85996284	0.00000000
C	-1.95653508	-0.82483147	0.00000000
C	-0.88063273	0.24776399	-0.00000001

C	-0.00207046	-0.86945310	0.00000001
C	1.36708784	-0.77255547	0.00000003
C	1.85319219	0.58351334	0.00000003
C	0.99854013	1.68951854	-0.00000002
C	-0.43333900	1.55135772	-0.00000004
H	-3.05096033	-0.85992322	-0.00000003
H	2.04634765	-1.62818504	0.00000006
H	2.93385680	0.75474054	0.00000007
H	1.43379584	2.69290914	-0.00000001
H	-1.08650764	2.42832219	-0.00000009

Compound **42**
E = -323.40691751 a.u. (all vibrational frequencies real)

C	1.24936255	-1.72042157	0.00000000
C	2.05142212	-0.59926950	0.00000000
C	0.84296827	0.32844257	0.00000000
C	0.00250330	-0.83508650	0.00000000
N	-1.30445296	-0.90438610	0.00000000
C	-1.87121269	0.36885718	0.00000000
C	-1.15928076	1.56613895	0.00000000
C	0.28296496	1.58331888	0.00000000
H	1.44277457	-2.79426741	0.00000000
H	3.13116703	-0.44221184	0.00000000
H	-2.96433500	0.38764147	0.00000000
H	-1.71584230	2.50690334	0.00000000
H	0.84504686	2.52075508	0.00000000

Compound **43**
E = -339.42672912 a.u. (all vibrational frequencies real)

N	-1.14070501	-1.80352088	0.00000000
C	-1.95003761	-0.76089026	0.00000000
C	-0.87452009	0.31126412	0.00000000
C	-0.00390592	-0.81516661	-0.00000002
N	1.29581760	-0.87441069	-0.00000004
C	1.82646355	0.41589613	-0.00000001
C	1.08485111	1.59758600	0.00000003
C	-0.35625444	1.58551528	0.00000003
H	-3.04384987	-0.78317930	0.00000001
H	2.91898220	0.46410100	0.00000000
H	1.62084288	2.55015394	0.00000007
H	-0.94134292	2.50921338	0.00000006

Compound **44**
E = -398.51285046 a.u. (all vibrational frequencies real)

C	-1.51328452	0.93940773	0.00000000
N	-1.76563611	-0.46292304	0.00000000
C	-0.37287368	-0.75649921	0.00000000
C	-0.00167217	0.61067322	0.00000000
C	1.31792433	1.02585340	0.00000000
C	2.28751460	-0.02051201	0.00000000
C	1.90566338	-1.37260123	0.00000000
C	0.54159277	-1.79570067	0.00000000
O	-2.24227879	1.89451836	0.00000000
H	-2.61305709	-1.01741400	0.00000000
H	1.61324796	2.07721907	0.00000000
H	3.35076918	0.23133652	0.00000000
H	2.68672678	-2.13744552	0.00000000
H	0.27079889	-2.85310911	0.00000000

Compound **45**

E = -398.44999347 a.u. (all vibrational frequencies real)

C	-0.80391089	2.05697993	0.00000000
C	-1.98431655	1.35514506	0.00000000
C	-1.19531548	0.02266230	0.00000000
C	0.00138375	0.74847182	0.00000000
C	1.30300536	0.15412213	0.00000000
N	1.13442482	-1.26690810	0.00000000
C	-0.01479944	-1.99580198	0.00000000
C	-1.26794240	-1.37408410	0.00000000
H	-0.53998152	3.11520958	0.00000000
H	-3.04206522	1.62101016	0.00000000
O	2.43334749	0.64138287	0.00000000
H	2.02480276	-1.76337221	0.00000000
H	0.10844975	-3.08140611	0.00000000
H	-2.18757656	-1.95911749	0.00000000

Table S3. CASPT2/cc-pVDZ Aromaticity Indices for the Benchmark Compounds in Figure 2[a]

Compound[b]	$NICS_{zz}(0)$[c]	$NICS_{zz}(1)$[c]	$NICS_{zz}(2)$[c]	MCI[c]	SA[c]	HOMA	HOMAc
**1**	-11.103	-26.884	-16.683	0.602	0.0000	0.912	0.968
**2**	-9.645	-26.389	-15.951	0.600	0.0008	0.944	0.974
**3**	-6.726	-25.280	-15.222	0.595	0.0033	0.942	0.971
**4**	-8.037	-25.792	-15.224	0.598	0.0008	0.971	0.979
**5**	-7.911	-25.604	-15.167	0.599	0.0007	0.960	0.974
**6**	-5.614	-24.826	-14.446	0.596	0.0000	0.988	0.982
**7**	6.978	-11.084	-9.570	0.487	0.0011	0.572	0.693
**8**	-8.602	-24.886	-15.182	0.591	0.0013	0.942	0.973
**9**	17.630	-1.342	-5.608	0.383	0.0011	0.485	0.564
**10**	-5.080	-21.978	-13.551	0.579	0.0015	0.972	0.982
**11**	18.385	-2.044	-6.022	0.412	0.0039	-0.132	0.255
**12**	17.536	-2.483	-5.939	0.385	0.0035	-0.187	0.359
**13**	-9.782	-25.342	-15.870	0.596	0.0000	0.901	0.962
**14**	-13.398	-28.569	-17.288	0.616	0.0000	0.899	0.961
**15**	93.412	41.783	5.733	0.306	0.0136	-2.812	-1.177
**16**	97.477	35.423	3.996	0.256	0.0192	-1.728	-1.250
**17**	117.188	35.549	4.099	0.249	0.0311	-0.892	-1.271
**18**	103.329	31.603	3.040	0.314	0.0221	-0.834	-1.212
**19**	42.309	7.218	-3.290	0.326	0.0052	-1.076	-0.465
**20**	80.078	29.058	2.038	0.284	0.0213	-1.569	-1.138
**21**	82.046	37.826	4.546	0.298	0.0135	-2.924	-1.243
**22**	90.012	40.073	5.308	0.288	0.0121	-2.556	-1.025
**23**	-11.101	-30.475	-15.633	0.545	0.0004	0.837	0.868
**24**	-13.215	-32.383	-15.666	0.544	0.0036	0.902	0.938
**25**	-11.366	-30.857	-15.047	0.544	0.0025	0.892	0.865
**26**	-4.666	-24.652	-13.149	0.494	0.0031	0.218	0.578
**27**	-5.272	-25.231	-12.780	0.493	0.0073	0.272	0.547
**28**	14.143	0.052	-5.251	0.458	0.0019	0.164	0.543
**29** (right)	-7.994	-25.112	-17.030	0.551	0.0004	0.697	0.852
**29** (left)	-7.994	-25.112	-17.030	0.551	0.0004	0.697	0.852
**30** (right)	-6.318	-24.556	-16.428	0.548	0.0004	0.761	0.864
**30** (left)	-8.958	-25.290	-16.939	0.550	0.0016	0.706	0.857
**31** (right)	-6.631	-24.554	-16.289	0.549	0.0004	0.765	0.870

**31** (left)	-7.836	-25.108	-16.992	0.551	0.0017	0.724	0.866
**32** (right)	-10.636	-27.470	-16.634	0.570	0.0010	0.936	0.960
**32** (left)	-3.690	-25.853	-14.766	0.479	0.0024	0.819	0.813
**33** (right)	-5.796	-23.170	-14.894	0.554	0.0011	0.938	0.960
**33** (left)	-3.634	-25.980	-14.653	0.489	0.0022	0.849	0.829
**34** (right)	13.288	-6.421	-8.523	0.432	0.0038	0.636	0.685
**34** (left)	-6.324	-27.303	-14.167	0.522	0.0022	0.876	0.858
**35** (right)	15.366	-4.641	-7.957	0.424	0.0045	0.649	0.684
**35** (left)	-4.665	-26.241	-13.763	0.519	0.0024	0.866	0.845
**36** (right)	19.938	0.770	-5.640	0.343	0.0010	0.536	0.575
**36** (left)	-3.173	-25.082	-13.223	0.518	0.0029	0.862	0.829
**37** (right)	11.663	-7.510	-8.343	0.459	0.0038	0.769	0.763
**37** (left)	-0.850	-13.743	-8.284	0.405	0.0005	0.744	0.684
**38** (right)	-4.664	-23.259	-15.419	0.546	0.0017	0.815	0.878
**38** (left)	-7.032	-24.348	-15.534	0.545	0.0017	0.846	0.897
**39** (right)	-5.768	-23.705	-14.617	0.581	0.0012	0.898	0.940
**39** (left)	19.302	-1.561	-6.808	0.393	0.0047	0.410	0.577
**40** (right)	83.259	33.233	2.534	0.345	0.0072	-1.579	-0.442
**40** (left)	8.020	-9.258	-8.599	0.562	0.0008	0.655	0.820
**41** (right)	89.745	30.399	1.510	0.294	0.0122	-0.875	-0.493
**41** (left)	2.965	-12.498	-9.799	0.570	0.0008	0.706	0.847
**42** (right)	75.058	29.562	1.840	0.351	0.0072	-1.617	-0.464
**42** (left)	10.353	-8.246	-7.928	0.554	0.0027	0.702	0.799
**43** (right)	80.663	26.567	0.783	0.338	0.0123	-0.881	-0.498
**43** (left)	6.073	-10.850	-8.914	0.561	0.0032	0.740	0.814
**44** (right)	48.603	10.635	-3.873	0.328	0.0040	-1.060	-0.408
**44** (left)	-6.093	-21.180	-13.836	0.582	0.0004	0.818	0.913
**45** (right)	93.386	42.751	5.533	0.318	0.0108	-2.103	-0.756
**45** (left)	9.663	-7.334	-6.488	0.489	0.0010	0.747	0.764

[a]$NICS_{zz}$ values in ppm. [b]For the polycyclic compounds **29**–**45**, aromaticity indices are given for the right-most and left-most rings as shown in Figure 2. [c]Obtained by means of CASSCF/cc-pVDZ calculations.

5. Complementary Correlations Between Aromaticity Indices (Figures S1–S8)

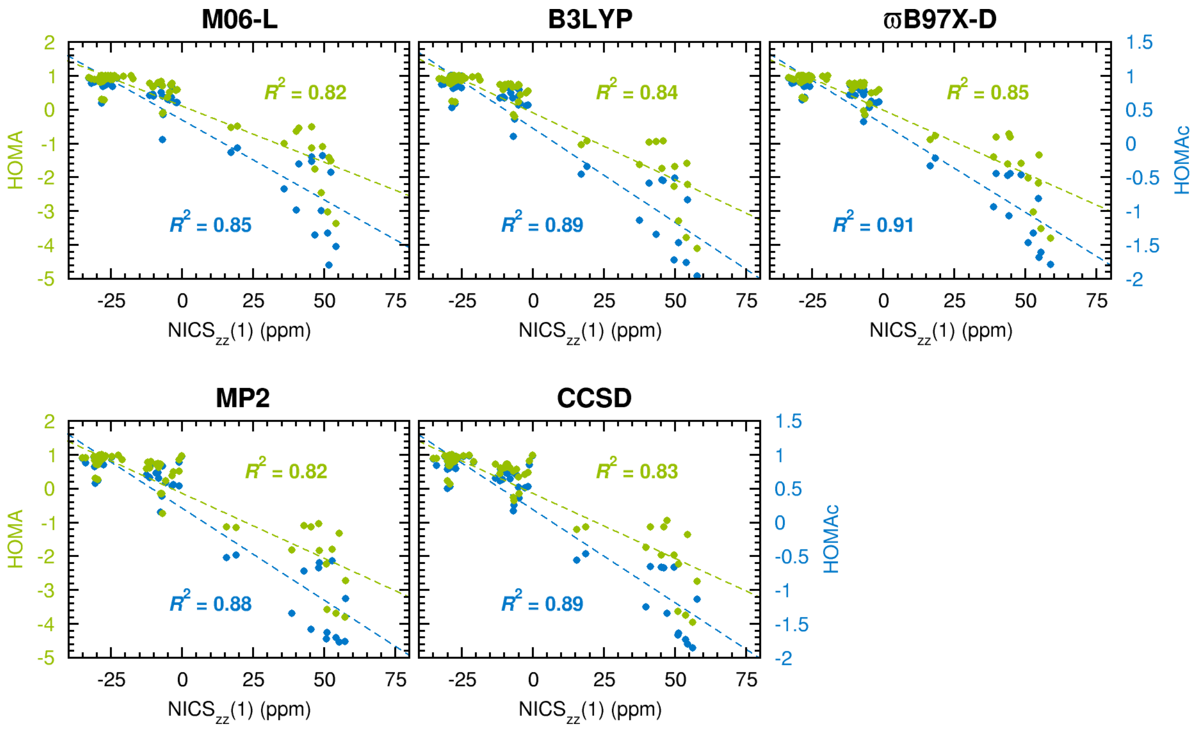

Figure S1. Linear correlations to NICS$_{zz}$(1) values achieved by HOMAc (blue font) and HOMA values (green font) calculated at the M06-L, B3LYP, ωB97X-D, MP2 and CCSD levels of theory.

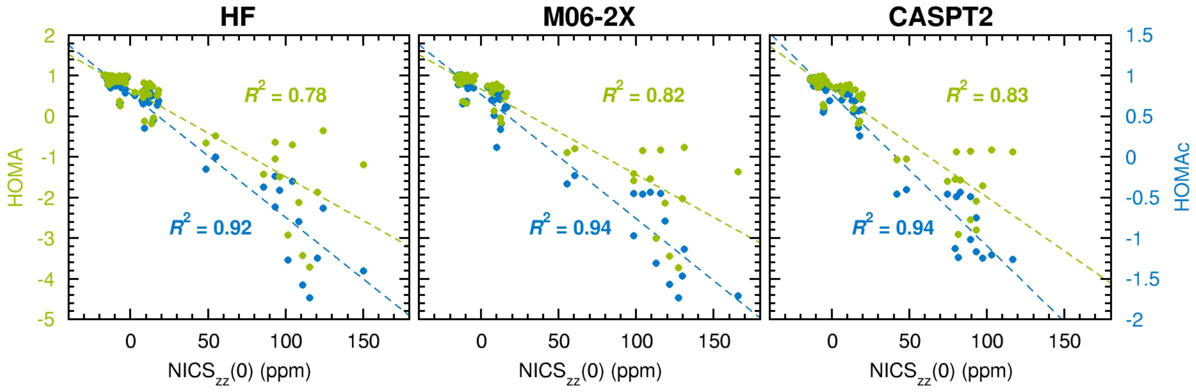

Figure S2. Linear correlations to NICS$_{zz}$(0) values achieved by HOMAc (blue font) and HOMA values (green font) calculated at the HF, M06-2X and CASPT2 levels of theory.

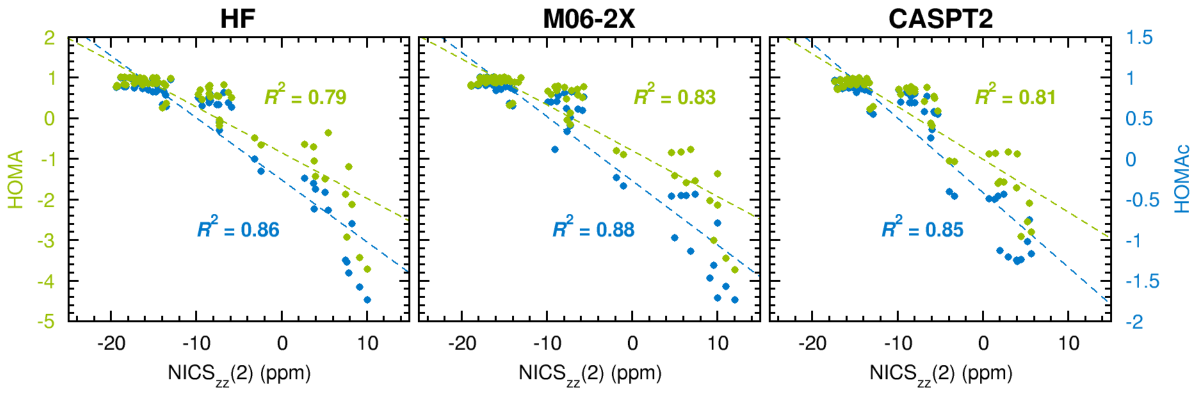

Figure S3. Linear correlations to NICS$_{zz}$(2) values achieved by HOMAc (blue font) and HOMA values (green font) calculated at the HF, M06-2X and CASPT2 levels of theory.

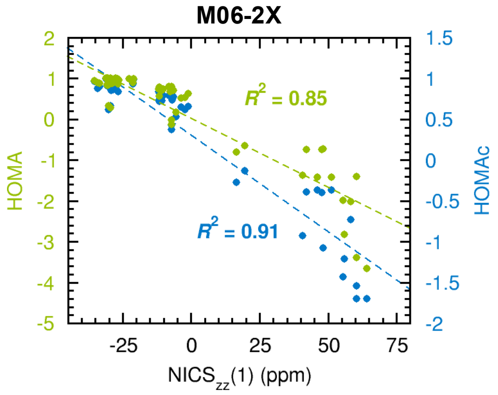

Figure S4. Linear correlations to NICS$_{zz}$(1) values achieved by HOMAc (blue font) and HOMA values (green font) calculated at the M06-2X level of theory, but using a larger basis set (cc-pVTZ instead of cc-pVDZ) than in all other calculations performed to compare HOMAc and HOMA.

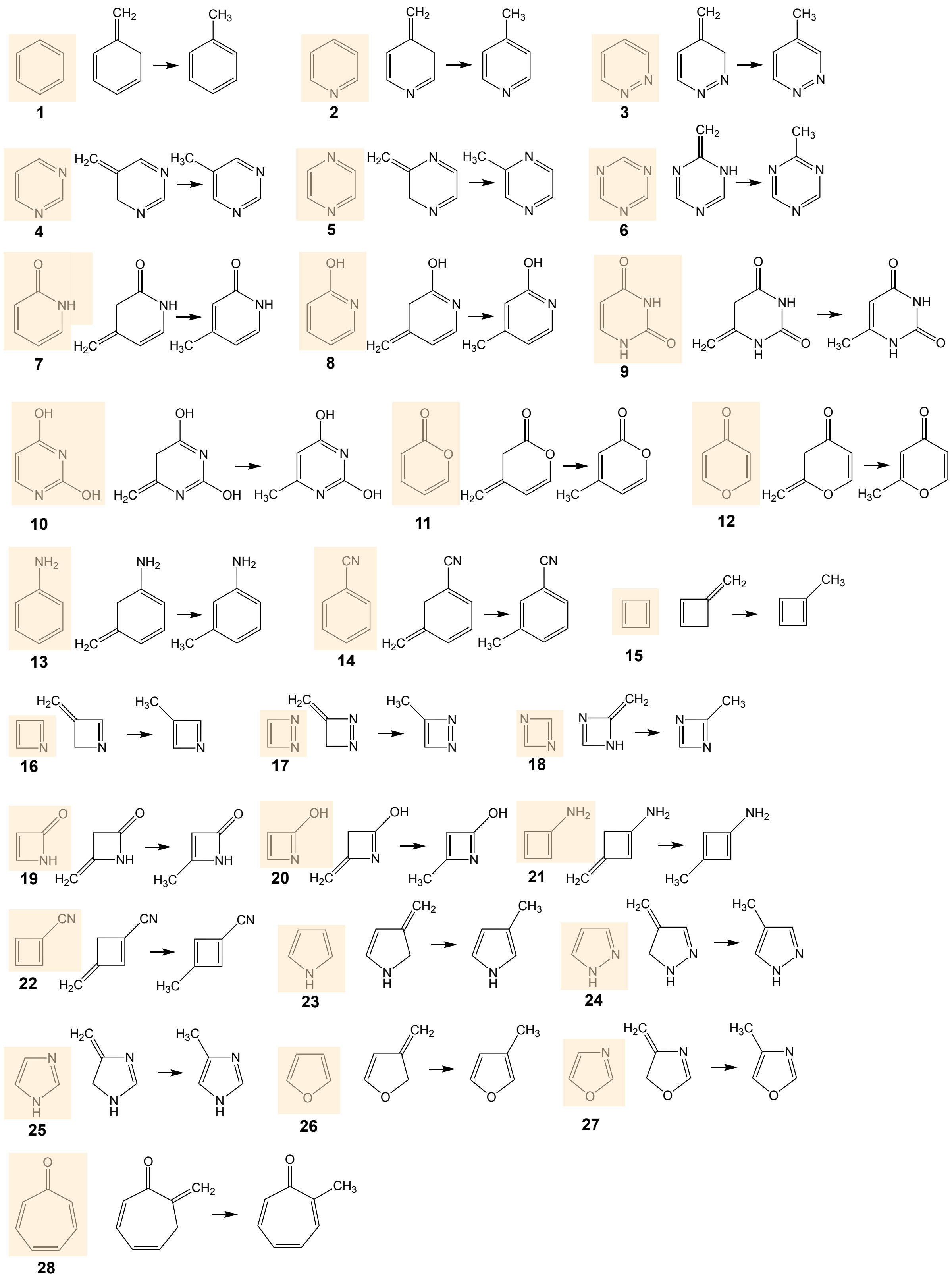

Figure S5. Isomeric pairs used to calculate the ISE values for the monocyclic benchmark compounds **1**–**28** given in Figure S6.

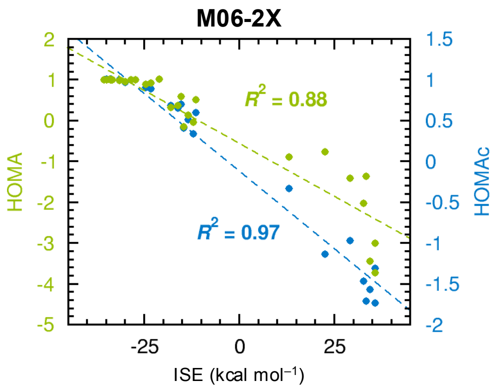

Figure S6. Linear correlations to ISE values achieved by HOMAc (blue font) and HOMA values (green font) calculated at the M06-2X level of theory for the monocyclic benchmark compounds **1**–**28**. The ISE values were obtained from electronic energies using the isomeric pairs shown in Figure S5, and do not include any correction terms to account for the *anti*-*syn* diene conformational mismatches within the isomeric pairs.

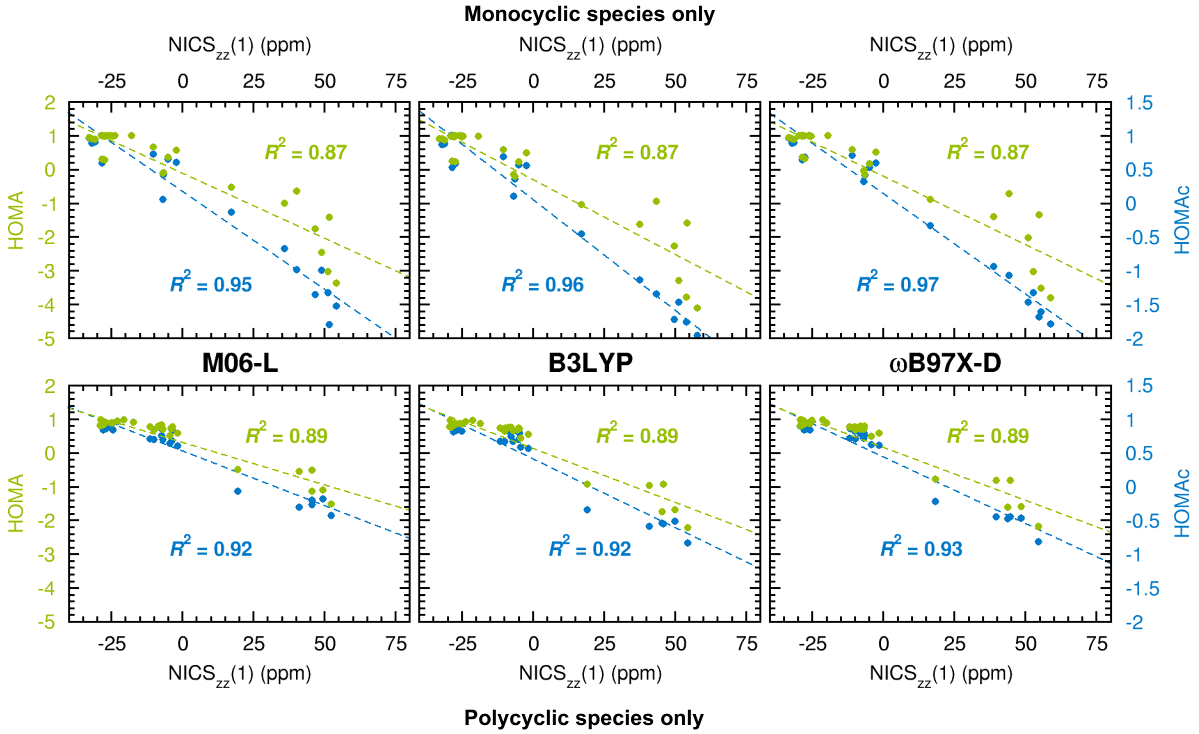

Figure S7. Linear correlations to NICS$_{zz}$(1) values achieved by HOMAc (blue font) and HOMA values (green font) calculated at the M06-L, B3LYP and ωB97X-D levels of theory, when the benchmark set of investigated compounds is divided into two subsets containing exclusively monocyclic (upper panels) or polycyclic (lower panels) species.

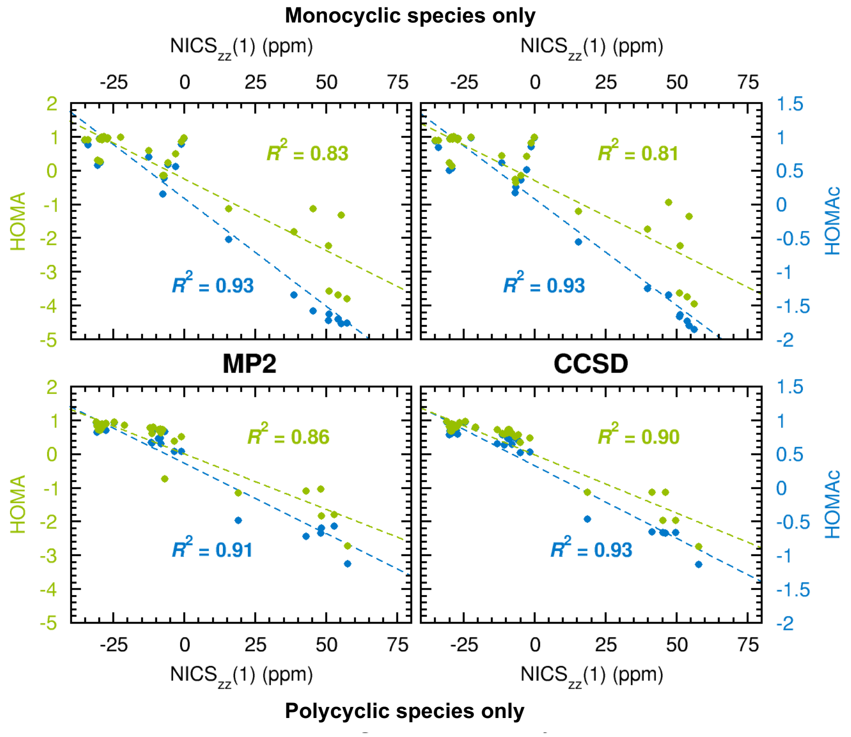

Figure S8. Linear correlations to NICS$_{zz}$(1) values achieved by HOMAc (blue font) and HOMA values (green font) calculated at the MP2 and CCSD levels of theory, when the benchmark set of investigated compounds is divided into two subsets containing exclusively monocyclic (upper panels) or polycyclic (lower panels) species.

6. Comparison of Linear and Quadratic Correlations Between Aromaticity Indices (Table S4)

Table S4. Comparison of Linear and Quadratic Correlations (LC and QC) with $NICS_{zz}(1)$, SA and MCI Values Achieved by HOMA and HOMAc Values Calculated at the HF, M06-2X and CASPT2 Levels of Theory[a]

Measure[b]	LC with $NICS_{zz}(1)$	QC with $NICS_{zz}(1)$	LC with SA	QC with SA	LC with MCI	QC with MCI
			**HOMA (HF/cc-pVDZ)**			
R^2	0.832	0.882	0.587	0.721	0.684	0.733
CI(*m*)	[-0.166, 0.079]		[0.648, 1.121]		[-3.866, -2.490]	
CI(*k*)	[-0.040, -0.031]		[-128.8, -82.9]		[5.398, 8.034]	
			**HOMAc (HF/cc-pVDZ)**			
R^2	0.910	0.954	0.783	0.841	0.791	0.885
CI(*m*)	[0.228, 0.344]		[0.852, 1.074]		[-2.295, -1.544]	
CI(*k*)	[-0.026, -0.022]		[-90.1, -68.6]		[3.992, 5.431]	
			**HOMA (M06-2X/cc-pVDZ)**			
R^2	0.868	0.908	0.621	0.783	0.714	0.756
CI(*m*)	[-0.111, 0.112]		[0.622, 1.079]		[-3.378, -2.029]	
CI(*k*)	[-0.038, -0.031]		[-128.7, -85.4]		[3.916, 6.160]	
			**HOMAc (M06-2X/cc-pVDZ)**			
R^2	0.911	0.932	0.825	0.883	0.862	0.889
CI(*m*)	[0.227, 0.352]		[0.833, 1.044]		[-2.046, -1.171]	
CI(*k*)	[-0.026, -0.022]		[-93.8, -73.9]		[2.808, 4.263]	
			**HOMA (CASPT2/cc-pVDZ)**[c]			
R^2	0.859	0.902	0.546	0.753	0.718	0.780
CI(*m*)	[-0.247, -0.030]		[0.538, 0.993]		[-4.319, -3.034]	
CI(*k*)	[-0.047, -0.038]		[-156.2, -96.7]		[6.843, 9.483]	
			**HOMAc (CASPT2/cc-pVDZ)**[c]			
R^2	0.892	0.919	0.793	0.868	0.816	0.914
CI(*m*)	[0.136, 0.266]		[0.802, 1.014]		[-2.760, -2.045]	
CI(*k*)	[-0.032, -0.027]		[-118.7, -91.0]		[5.248, 6.718]	

[a]$NICS_{zz}(1)$ values in ppm. [b]The confidence intervals (CI) for the linear-regression parameters ($y = m + kx$) are given at 95% confidence level. [c]The HOMA and HOMAc values calculated at the CASPT2/cc-pVDZ level are correlated with $NICS_{zz}(1)$, SA and MCI values calculated at the CASSCF/cc-pVDZ level.

7. Calculated Aromaticity Indices for Other Species (Table S5)

Table S5. M06-2X/cc-pVDZ Aromaticity Indices for Other Species[a]

Species	$NICS_{zz}(1)$	HOMA	HOMAc
Non-aromatic saturated compounds			
Cyclohexane	–[b]	-4.19	-1.92
Cyclobutane	–[b]	-5.82	-2.86
Cyclohexylamine	–[b]	-4.09	-1.86
Cyclobutylamine	–[b]	-5.27	-2.54
Cyclohexanecarbonitrile	–[b]	-4.35	-2.01
Cyclobutanecarbonitrile	–[b]	-5.61	-2.74
Strongly antiaromatic transition-state geometries			
C_4H_4 (D_{4h} symmetry	79.94	0.25	0.62
$C_2N_2H_2$ (D_{2h} symmetry	100.26	0.66	0.58
N_4 (D_{4h} symmetry)	57.72	0.45	0.69
C_8H_8 (D_{4h} symmetry)	90.56	-0.30	0.25
C_8H_8 (D_{8h} symmetry)	137.78	0.92	0.97

[a] $NICS_{zz}(1)$ values in ppm. [b] $NICS_{zz}(1)$ value not calculated since non-planar geometry.

8. Active Spaces in CASSCF and CASPT2 Calculations (Table S6)

Table S6. Active Spaces Comprising π and π* Orbitals Used in CASSCF and CASPT2 Calculations

Compound	Active space	Compound	Active space	Compound	Active space
**1**	(6,6)	**16**	(4,4)	**31**	(10,10)
**2**	(6,6)	**17**	(4,4)	**32**	(10,9)
**3**	(6,6)	**18**	(4,4)	**33**	(10,9)
**4**	(6,6)	**19**	(4,4)	**34**	(10,9)
**5**	(6,6)	**20**	(4,4)	**35**	(10,9)
**6**	(6,6)	**21**	(4,4)	**36**	(10,9)
**7**	(6,6)	**22**	(4,4)	**37**	(12,10)
**8**	(6,6)	**23**	(6,5)	**38**	(10,10)
**9**	(6,6)	**24**	(6,5)	**39**	(10,10)
**10**	(6,6)	**25**	(6,5)	**40**	(8,8)
**11**	(6,6)	**26**	(6,5)	**41**	(8,8)
**12**	(6,6)	**27**	(6,5)	**42**	(8,8)
**13**	(6,6)	**28**	(8,8)	**43**	(8,8)
**14**	(6,6)	**29**	(10,10)	**44**	(8,8)
**15**	(4,4)	**30**	(10,10)	**45**	(8,8)
